# The Lack of Alternative Oxidase 1a Restricts *in vivo* Respiratory Activity and Stress-Related Metabolism for Leaf Osmoprotection and Redox Balancing Under Sudden Acute Water and Salt Stress in *Arabidopsis thaliana*

**DOI:** 10.3389/fpls.2022.833113

**Published:** 2022-05-17

**Authors:** Néstor F. Del-Saz, Ariadna Iglesias-Sanchez, David Alonso-Forn, Miguel López-Gómez, Francisco Palma, María José Clemente-Moreno, Alisdair R. Fernie, Miquel Ribas-Carbo, Igor Florez-Sarasa

**Affiliations:** ^1^Laboratorio de Fisiología Vegetal, Universidad de Concepción, Concepción, Chile; ^2^Centre for Research in Agricultural Genomics CSIC-IRTA-UAB-UB, Barcelona, Spain; ^3^Unidad de Recursos Forestales, Centro de Investigación y Tecnología Agroalimentaria de Aragón, Zaragoza, Spain; ^4^Department of Plant Physiology, University of Granada, Granada, Spain; ^5^Grup de Recerca en Biologia de les Plantes en Condicions Mediterranies, Departament de Biologia, Universitat de les Illes Balears, Palma, Spain; ^6^Max Planck Institute of Molecular Plant Physiology, Potsdam, Germany; ^7^Institut de Recerca i Tecnología Agroalimentàries (IRTA), Edifici CRAG, Barcelona, Spain

**Keywords:** water stress, salinity, alternative oxidase, oxygen-isotope fractionation, primary metabolism, photosynthesis, *Arabidopsis thaliana*

## Abstract

In plants salt and water stress result in an induction of respiration and accumulation of stress-related metabolites (SRMs) with osmoregulation and osmoprotection functions that benefit photosynthesis. The synthesis of SRMs may depend on an active respiratory metabolism, which can be restricted under stress by the inhibition of the cytochrome oxidase pathway (COP), thus causing an increase in the reduction level of the ubiquinone pool. However, the activity of the alternative oxidase pathway (AOP) is thought to prevent this from occurring while at the same time, dissipates excess of reducing power from the chloroplast and thereby improves photosynthetic performance. The present research is based on the hypothesis that the accumulation of SRMs under osmotic stress will be affected by changes in folial AOP activity. To test this, the oxygen isotope-fractionation technique was used to study the *in vivo* respiratory activities of COP and AOP in leaves of wild-type *Arabidopsis thaliana* plants and of *aox1a* mutants under sudden acute stress conditions induced by mannitol and salt treatments. Levels of leaf primary metabolites and transcripts of respiratory-related proteins were also determined in parallel to photosynthetic analyses. The lack of *in vivo* AOP response in the *aox1a* mutants coincided with a lower leaf relative water content and a decreased accumulation of crucial osmoregulators. Additionally, levels of oxidative stress-related metabolites and transcripts encoding alternative respiratory components were increased. Coordinated changes in metabolite levels, respiratory activities and photosynthetic performance highlight the contribution of the AOP in providing flexibility to carbon metabolism for the accumulation of SRMs.

## Introduction

Plants display several mechanisms for drought and salinity tolerance, however, these come with high energy and carbon demands that plant metabolism meets by adjusting photosynthesis and respiration (Munns et al., 2020; Vanlerberghe et al., 2020). An important tolerance mechanism to drought and salinity resides in the accumulation of metabolites that play multiple protective roles, including primary metabolites such as organic acids, sugars and polyols (Sanchez et al., 2008; Fàbregas and Fernie, 2019), as well as polyamines (Alcázar et al., 2020). These stress-related metabolites (SRMs) from primary metabolism, protect against osmotic and oxidative stress, serve as energy sources and/or are involved in stress-signalling pathways (Slama et al., 2015; Pires et al., 2016; Fàbregas and Fernie, 2019). At the same time, the synthesis and degradation of several SRMs involve metabolic pathways that are tightly linked to respiratory metabolism (Pires et al., 2016; Batista-Silva et al., 2019; Bandehagh and Taylor, 2020). Thus, SRMs or their carbon precursors [i.e., glycolytic or tricarboxylic acid (TCA) cycle intermediates] are tightly associated to the generation of reducing equivalents (NADH and FADH_2_), from which oxidation is coupled to the mitochondrial electron transport chain (mETC). A distinctive feature of the plant mETC is the presence of two terminal oxidases. In addition to the cyanide sensitive cytochrome oxidase (COX), the presence of a cyanide-resistant alternative oxidase (AOX) that couples the oxidation of ubiquinol with the reduction of O_2_ to H_2_O, confers to mETC the capacity to sustain O_2_ consumption under COX restriction (Del-Saz et al., 2018; Vanlerberghe et al., 2020). Electron transport via the AOX pathway (AOP) is not coupled with energy conservation (Del-Saz et al., 2018; Vanlerberghe et al., 2020). Nonetheless, the AOP provides metabolic flexibility that helps plants acclimate to stress by allowing the oxidation of reducing equivalents coupled to TCA cycle and/or cytosolic reactions under conditions where the COX pathway (COP) is restricted (Del-Saz et al., 2018). In fact, the accumulation of SRMs has been reported to coincide with higher respiratory rates (Mattoo et al., 2006; Del-Saz et al., 2016; Pires et al., 2016; Bandehagh and Taylor, 2020), indicating an active mitochondrial metabolism under stress. Nevertheless, direct evidence about the influence of the AOP activity in modulating the synthesis of stress-related metabolites remains scarce.

The AOX protein is encoded by a multigene family which exhibits tissue-specific expression (Saisho et al., 1997; Clifton et al., 2006; Selinski et al., 2018a). AOX1 isoforms generally show the highest expression levels, being expressed in most tissues and induced by stress treatments in several species (Clifton et al., 2006; Vanlerberghe et al., 2020). Over the last two decades, reverse genetics has been used to decrease *AOX1* gene expression in order to test hypotheses regarding the role of the AOP. Following the suppression of *AOX1*, the amount of the AOX protein has been found to be markedly decreased concomitantly with a decrease in the AOP capacity (the maximum possible flux of electrons to AOX; Guy and Vanlerberghe, 2005; Umbach et al., 2005; Watanabe et al., 2010; Florez-Sarasa et al., 2011; Xu et al., 2012; Vishwakarma et al., 2015; Zhang et al., 2016; Garmash et al., 2020). In parallel, many studies have observed an increase in AOX protein or capacity under stress in wild-type plants (reviewed in Vanlerberghe, 2013; and in Vanlerberghe et al., 2020). In fact, a regulated overcapacity of AOP is thought to be required for ensuring an adequate activity under conditions inducing metabolic fluctuations (Rasmusson et al., 2009; Florez-Sarasa et al., 2011). By contrast to AOP capacity, *in vivo* AOP activity can be directly determined in the absence of added inhibitors by measuring the differential oxygen isotope discrimination during respiration (Day et al., 1996; Del-Saz et al., 2017). While there is substantial evidence on the molecular mechanisms regulating the AOX gene expression in response to drought and salinity stresses (Vanlerberghe, 2013; Dahal and Vanlerberghe, 2017; Zhu et al., 2018; Gong et al., 2020), only some studies have addressed when and to which extent the *in vivo* AOP activity is effectively responding. The *in vivo* AOP activity was reported to increase in response to severe drought and salt stress (Ribas-Carbo et al., 2005; Del-Saz et al., 2016), while unaltered activities were reported under milder or longer drought/salinity periods (Galle et al., 2010; Martí et al., 2011). These different responses denote, as previously highlighted (Munns et al., 2020), that *in vivo* AOP activity determinations in combination with detailed metabolic and molecular analyses remain crucial for understanding the key changes in energy and carbon metabolism conferring drought (Vanlerberghe et al., 2020) and salt (Munns et al., 2020) tolerance.

In order to gain insight into the role of the AOP during stress-related metabolism, wild-type *Arabidopsis thaliana* plants and *aox1a* T-DNA insertional mutants, from which information regarding *in vivo* activity is still lacking, were treated with high concentrations (1 day, 300 mM) of mannitol and salt (NaCl), known to greatly induce the accumulation of several SRMs (Del-Saz et al., 2016; Darko et al., 2019). The *in vivo* activities of COP and AOP in leaves were measured using the oxygen-isotope discrimination technique and in parallel, leaf levels of primary metabolites and respiratory-related transcripts were also determined. In addition, leaf relative water content as well as photosynthetic performance, determined by chlorophyll fluorescence analysis in the dark and in the light, was also studied. We hypothesized that the lack of *AOX1a* will be associated with lower leaf rates of AOP respiration and less accumulation of SRMs under severe water and salt stress, with consequences for plant performance under stress.

## Materials and Methods

### Plant Material and Growth Conditions

Seeds of wild-type (WT) *A. thaliana* Columbia 0 (Col 0) and *aox1a* T-DNA insertion line (SALK_084897) were sown in pre-wetted pots with a mixture of peat-perlite (3:1). The T-DNA insertion within the *AtAOX1a* gene was confirmed by genomic PCR with T-DNA-specific primers and seeds from a homozygous line were used for the experiments. Pots were placed in plastic trays used for sub-irrigation. Plants were grown in a growth chamber for 32–35 days at 12 h/12 h photoperiod, 150 μmol quanta m^–2^ s^–1^ light intensity, 25°C/20°C day/night temperature and relative humidity above 50%. Plants were watered twice a week with half-strength Hoagland’s solution (Epstein et al., 1972). On days 32–35, plants were divided in three groups, one was watered normally (control plants) while the other two were irrigated with watering solution containing 300 mM of sodium chloride (NaCl-treated plants) or 300 mM mannitol (Mannitol-treated plants). The analyses of control and treated plants were performed one day after the irrigation. All the experiments were repeated at least twice, and data is presented as a pool of at least two individual experiments (i.e., each consisting of two to four biological replicates/plants per genotype and treatment), since similar results were obtained in each individual experiment.

### Respiration and Oxygen-Isotope Fractionation Measurements

Leaves were placed in the dark for 30 min to avoid light-enhanced dark respiration. The capacity of the alternative pathway (V_alt_) was determined with an oxygen electrode as previously described (Del-Saz et al., 2016), and results were expressed in nmol O_2_ g^–1^ dry weight s^–1^ as the mean ± SE of five or six biological replicates. Respiration and oxygen isotope fractionation measurements were performed as previously described (Del-Saz et al., 2016). Respiratory partitioning between the two respiratory pathways was calculated from the oxygen isotope fractionation by the alternative oxidase (Δ_a_) and the cytochrome oxidase (Δ_c_). The Δ_a_ values, determined in the presence of 10 mM of KCN, were not significantly different between the genotypes and thus, the Δ_a_ mean value of 30.8 ± 0.445‰ (six replicates) was used for all the partitioning calculations. The Δ_c_, determined in presence of 25 mM of salicylhydroxamic acid (SHAM), was 20.5‰ (four replicates). Dry weights were obtained after drying leaves for 2 days at 60–70°C. Results were expressed in nmol O_2_ g^–1^ dry weight s^–1^ as the mean ± SE of six or eight biological replicates.

### Leaf Water Status

Leaf relative water content (RWC) was determined as follows:


RWC(%)=(FW-DW)/(TW-DW)× 100

where fresh (FW), turgid (TW) and dry (DW) weight of leaves were measured. FW was determined immediately after sampling; TW was obtained after incubating leaf discs in distilled water for 48 h in the dark at 4 °C (to minimize respiration losses) and DW was determined after drying for at least 2 days in an oven at 60–70°C.

### Photosynthetic Measurements

Chlorophyll fluorescence measurements were carried out on rosette leaves using a MAXI-PAM fluorometer (Heinz Walz GmbH) as previously described (Morelli et al., 2021). Briefly, for every measurement six rosette leaves from each plant were considered. The actual quantum efficiency of the photosystem II (PSII)-driven electron transport (ΦPSII) was determined under different light intensities (in μmol photons m^–2^ s^–1^ of photon flux density, PPFD): 800, 700, 600, 500, 400, 300, 200, 150, 100, 75, 50, 25 and 0. The chloroplast electron transport rate (ETR) was calculated as the product of ΦPSII x PPFD x 0.84 x 0.5, where 0.84 and 0.5 were assumed values for the leaf light absorptance and the fraction of absorbed quanta available for PSII. From the light response curves, the associated parameters ETR_m_ (maximum electron transport rate) and alpha (photosynthetic rate in light limited region of the light curve) were characterized by fitting iteratively the model of the rETR versus E curves using MS Excel Solver (Platt and Gallegos, 1980). The fit was very good in all the cases (*r* > 0.98). After the light curves, the maximum quantum efficiency of PSII, F_v_/F_m_, was calculated as (F_m_ – F_o_)/F_m_, where F_m_ and F_o_ are the maximum and the minimum fluorescence of dark-adapted samples, respectively. For dark acclimation, plants were incubated for at least 30 min in darkness to allow the full relaxation of photosystems.

### Metabolite Profiling

Metabolite extractions were performed as described previously (Lisec et al., 2006) using approximately 50 mg of leaf tissue, previously frozen-powdered. Derivatization and GC–TOF–MS analyses were carried out as described previously (Lisec et al., 2006). Metabolites were identified manually by TagFinder software (Luedemann et al., 2012) using the reference library mass spectra and retention indices housed in the Golm Metabolome Database;^1^ (Kopka et al., 2005). The parameters used for the peak annotation of the 46 metabolites can be found in Supplementary Table 1, which follows previously reported recommendations (Fernie et al., 2011).

To process the GC-TOF-MS results, the intensity of a selected unique ion shown in Supplementary Table 1 was normalized to that of ribitol which was added to each sample as an internal standard, as well as to the fresh weight of the materials used for metabolite extraction. Fresh weight was then corrected by dry weight/fresh weight ratios used to determine the leaf RWC (see Section “Leaf Water Staus”). Thereafter, data were normalized to the mean value of Col-0 wild-type (WT) plants under control conditions (i.e., the value of all metabolites for WT at control conditions was set to 1). This normalization allowed the comparison of the relative metabolite levels among genotypes and treatments (Supplementary Table 2). In addition, leaf absolute metabolite levels (μmol gDW^– 1^) were also determined for all the metabolites detected and present in the authentic standards mixture (Supplementary Table 3). The experimental samples and standards mixtures containing 0, 100, 200, 500, and 1,000 ng of each compound were analysed in the same GC- MS runs, and the absolute metabolite amounts in the samples were calculated by extrapolation from standard curves using the peak intensity of a representative mass fragment. Values presented for both relative and absolute levels are means ± SE of four to six replicates corresponding to several fully expanded rosette leaves.

### Western Blot Analysis

**FIGURE 1:**
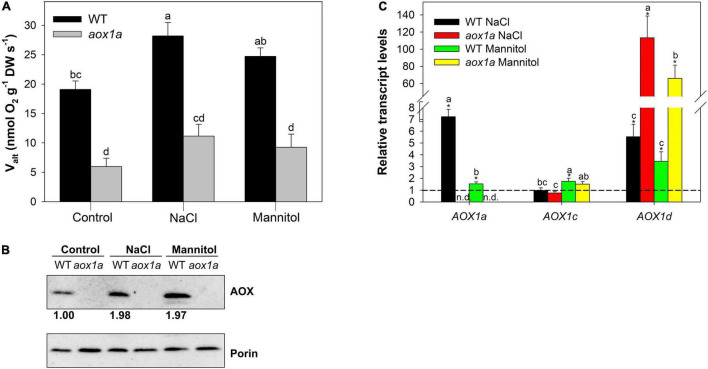
Alternative oxidase (AOX) pathway capacity (V_alt_) and expression analysis. **(A)** V_alt_ determined (see section “Materials and Methods”) in leaves of wild-type Col 0 (WT) and *aox1a Arabidopsis thaliana* plants untreated with NaCl or Mannitol (Control), and after 1 day of severe NaCl or Mannitol treatments (300 mM). Values are means ± SE of 5–6 biological replicates. Significant differences (*P* < 0.05) are denoted by different letters. **(B)** Western blot analysis of AOX and porin (VDAC) protein levels from total leaf protein extracts (see section “Material and Methods” for details). The intensities of the signals from AOX were normalized to those from porin (VDAC) and are expressed relative to WT levels under control conditions. The normalized intensity values shown are averages of three independent blots. **(C)** Transcript levels of genes encoding AOX isoforms determined by qPCR analyses (see Materials and Methods for details) under control and stress conditions. Data are shown as fold-changes relative to control conditions (i.e., all control values were set to 1, which is denoted by the dashed line). Primers used and gene information can be found in Supplementary Table 4. Values are means ± SE of 5–6 replicates and asterisks denote significant differences (*P* < 0.05) to the control condition for each gene expression analysis. Letters denote significant differences (*P* < 0.05) among genotypes and stress conditions for each gene expression analysis. n.d. (not detected).

Protein extraction and western blot analysis was performed as previously described (Florez-Sarasa et al., 2016) with some modifications. Total plant protein extracts were obtained from 20 mg of frozen leaf powder resuspended in 100 μl SDS sample buffer [2% (w/v) SDS, 62.5mM Tris–HCl (pH 6.8), 10% (v/v) glycerol and 0.007% (w/v) bromophenol blue], 50 mM DTT and protease inhibitor cocktail (Roche Basel, Switzerland). Samples were incubated 30 min at 4°C to allow full reduction of the AOX protein and then boiled (95°C) for 5 min. The resuspended sample was centrifuged at 14,000 rpm for 10 min. After loading 25 μl on a 12% SDS-PAGE gel, proteins were transferred to a 0.45 μm Nitrocellulose membrane (Armersham Protan) using a semi-dry Trans-Blot^®^ Turbo™ Transfer System of Bio-Rad. Membranes were incubated overnight at 4°C with diluted 1:500 polyclonal anti-AOX, AOX1 and 2 (AS04054, Agrisera, Sweden) or diluted 1:5,000 anti-Porin, voltage-dependent anion-selective channel protein 1–5 (AS07212, Agrisera, Sweden). Incubation with 1:20,000 horseradish peroxidase secondary antibodies (Cytiva) during 1h at room temperature. The detection of immunoreactive bands was performed using ECL SuperSignal™ West Femto Maximum Sensitivity Substrate (Thermo Fisher). Chemiluminescent signals were collected by IQ 800 Armersham (Cytiva). The protein band quantifications were performed with ImageJ analysis software according to the manufacturer’s instructions. The obtained band intensities for AOX were corrected for their corresponding porin band intensities and then normalized to the levels of the WT plants under control conditions (i.e., WT levels were set to 1). Three different immunoblot experiments per protein were performed and the image shown in Figure 1B belong to one of the three membranes obtained. Samples used in each of the three western blot experiments were a mixture of aliquots belonging to two biological replicates from the same six biological replicates used for the gene expression analysis. The relative values presented in Figure 1B are means of the three western blot experiments.

### Gene Expression Analysis

RNA was isolated from lyophilized leaves by using Maxwell^®^ RSC Plant RNA Kit (Promega Biotech Ibérica, Madrid, Spain) and automated system Maxwell^®^ RSC Instrument (Promega Biotech Ibérica, Madrid, Spain) according to the manufacturer’s instructions, and was quantified using a NanoDrop 1000 spectrophotometer (Thermo Scientific,^2^). Afterward cDNA synthesis was performed following the recommendations of the Transcriptor First Strand cDNA Synthesis Kit (Roche Basel, Switzerland). Relative mRNA abundance was evaluated by quantitative PCR using LightCycler 480 SYBR Green I Master Mix (Roche Basel, Switzerland) on a LightCycler 480 real-time PCR system (Roche Basel, Switzerland). Primers used and the related information are detailed in the Supplementary Table 4. Two technical replicates of each biological replicate were performed, and the mean values were used for further calculations. Arabidopsis *AtUbqC* (At5g25760) was used as a reference gene to correct for differences in the total amount of transcripts and the 2^–ΔΔCt^ method (Livak and Schmittgen, 2001) was used to calculate the fold-change of gene expression. Finally, data were normalized to the mean value of plants under control conditions in each genotype (i.e., the level of all transcripts for WT and *aox1a* mutants at control was set to 1).

### Statistical Analyses

**FIGURE 2:**
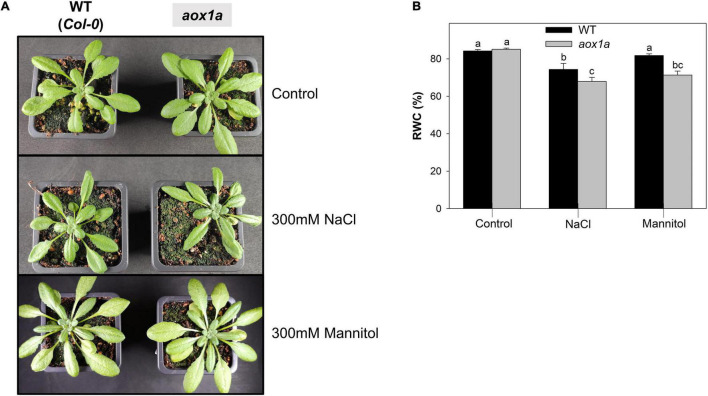
Visual phenotype and relative water content. **(A)** Photographs representative of WT and *aox1a* plants untreated with NaCl or Mannitol (Control), and after 1 day of severe NaCl or Mannitol treatments (300 mM). **(B)** Leaf relative water content (RWC) in leaves of WT and *aox1a* plants under control and stress conditions. Values are means ± SE of 10–12 biological replicates. Significant differences (*P* < 0.05) are denoted by different letters.

**FIGURE 3:**
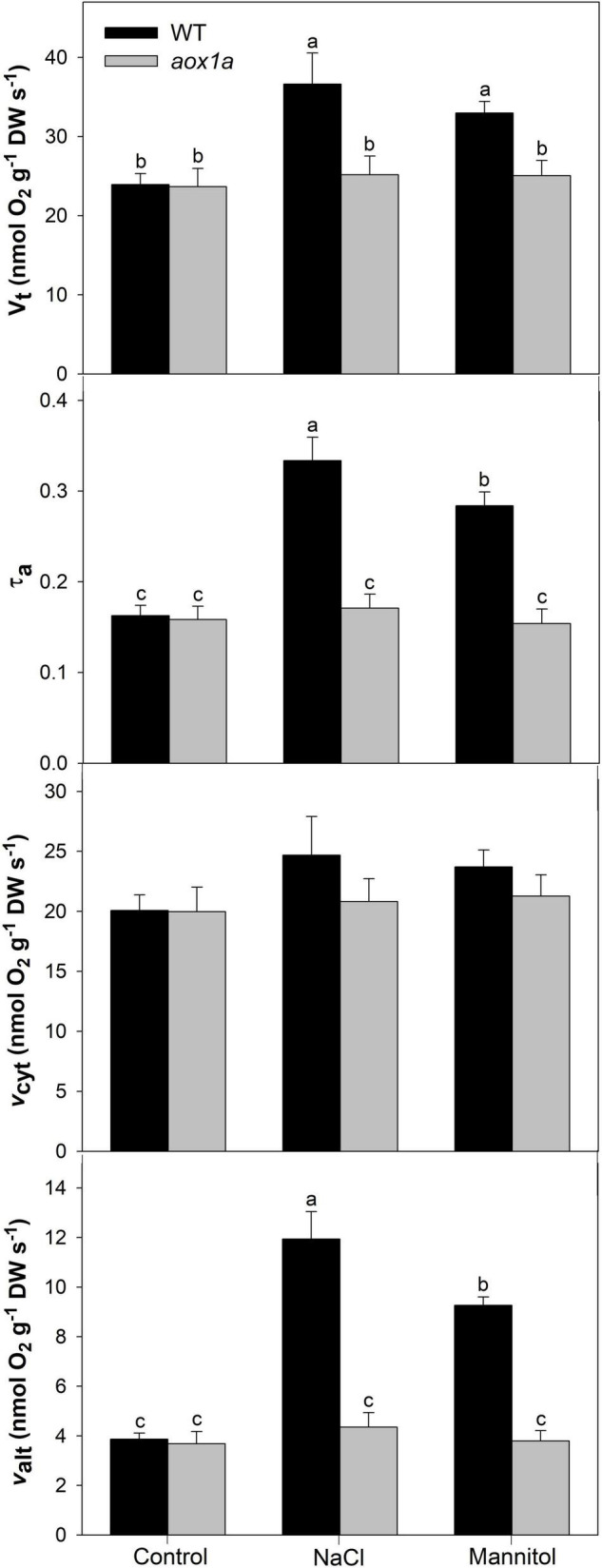
Respiration and electron partitioning between the cytochrome and the alternative oxidase pathways (COP and AOP). Total respiration (V_t_), electron partitioning to the AOP (τ_a_), COP activity (v_cyt_) and AOP activity (v_alt_) in leaves of Col 0 (WT) and *aox1a A. thaliana* plants untreated with NaCl or Mannitol (Control), and after 1 day of severe NaCl or Mannitol treatments (300 mM). Values are means ± SE of 6–8 biological replicates. Significant differences (*P* < 0.05) are denoted by different letters.

**FIGURE 4:**
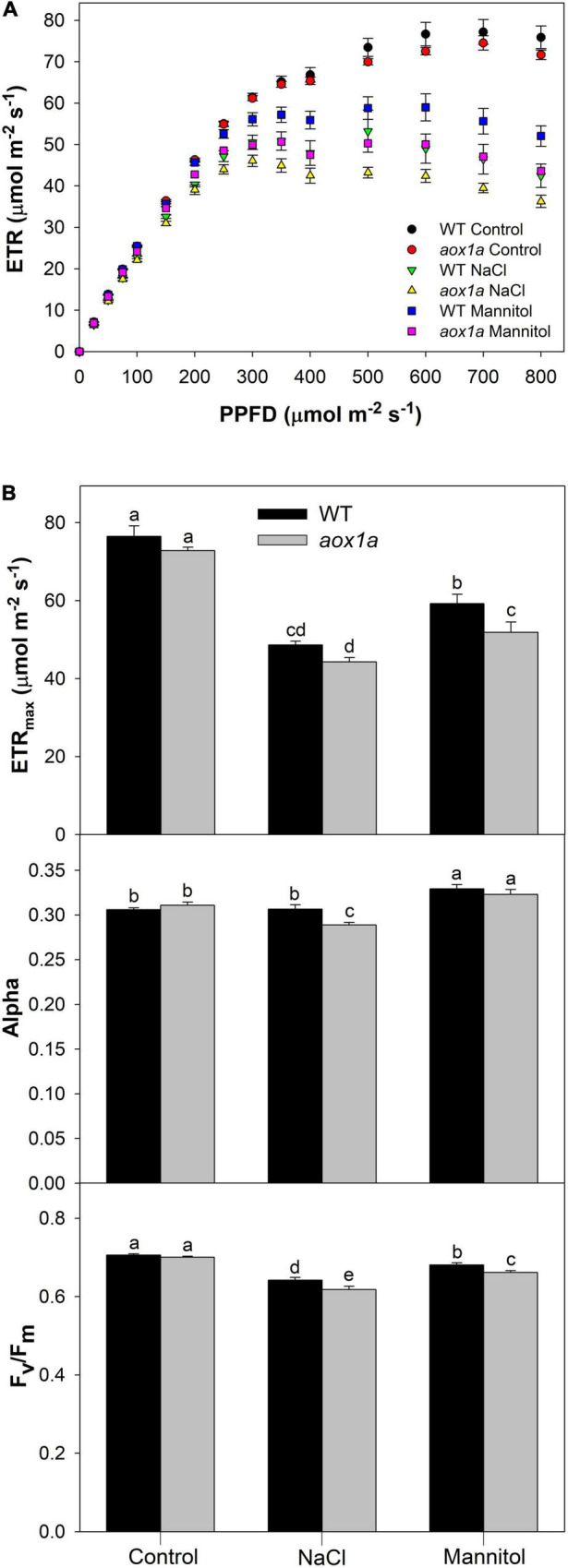
Photosynthetic activity and capacity under different light intensities. **(A)** Light curves showing light dependent response (see Materials and Methods for details) of the chloroplast electron transport rate (ETR) in leaves of wild-type Col 0 (WT) and *aox1a A. thaliana* plants untreated with NaCl or Mannitol (Control), and after 1 day of severe NaCl or Mannitol treatments (300 mM). Values are means ± SE of 5–6 biological replicates. **(B)** Maximum electron transport rate (ETR_max_) and photosynthetic rate in light limited region (alpha) were derived from light curves (see Materials and Methods for details) performed under control and stress conditions. The maximum quantum efficiency of PSII (Fv/Fm) was determined after 30 min in darkness (see Materials and Methods for details) under control and stress conditions. Values are means ± SE of 5–6 biological replicates. Significant differences (*P* < 0.05) are denoted by different letters.

**FIGURE 5:**
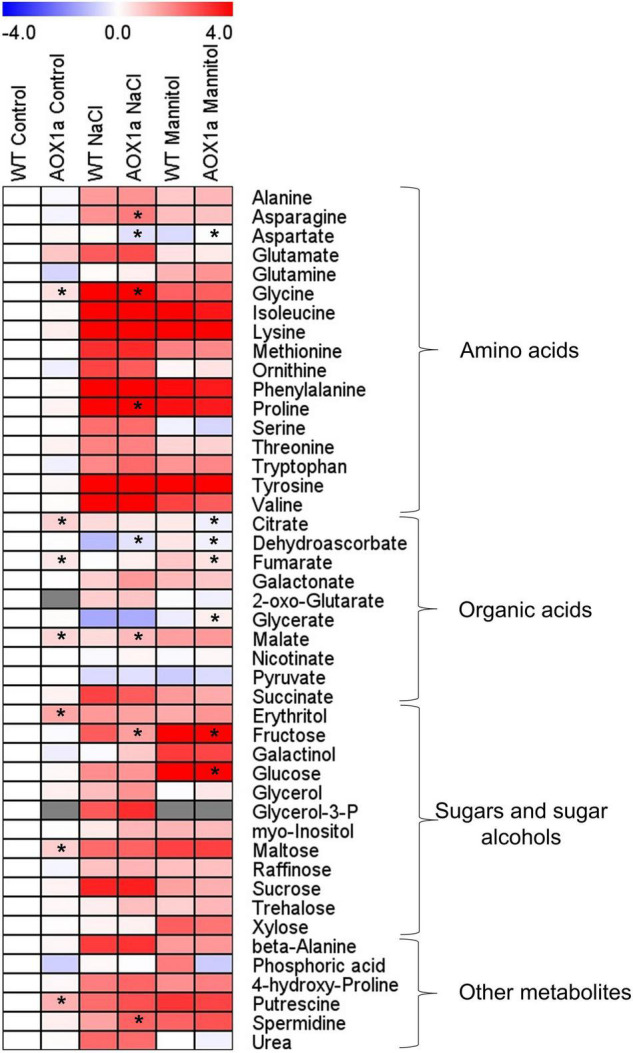
Metabolite profiles showing relative changes in primary metabolism. Heat map showing the relative levels of the GC-MS-analysed metabolites in leaves of wild-type Col 0 (WT) and *aox1a A. thaliana* plants untreated with NaCl or Mannitol (Control), and after 1 day of severe NaCl or Mannitol treatments (300 mM). Metabolites were clustered per class into amino acids, organic acids, sugars and sugar alcohols, and other metabolites. Relative metabolite levels were normalized to the mean level of the WT plants under control conditions and fold-change values were log2 transformed (i.e., the level of all metabolites of WT plants under control conditions is 0). In this heat map, red and blue colours represent log2 fold-increased and -decreased metabolites, respectively. Gray colours indicate the metabolites not detected in some experimental conditions. Values are means ± SE of 4–6 replicates and asterisks denote significant differences (*P* < 0.05) to the WT plants in each experimental condition. The statistical differences between control and stress treatments in each genotype are presented in Supplementary Table 2.

**FIGURE 6:**
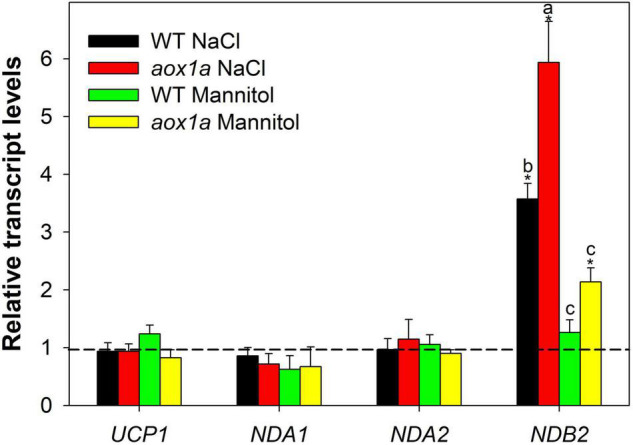
Gene expression analyses of alternative respiratory components. Transcript levels of genes encoding uncoupling proteins and alternative NAD(P)H dehydrogenases were determined by qPCR analyses (see Materials and Methods for details) in leaves of wild-type Col 0 (WT) and *aox1a A. thaliana* plants untreated with NaCl or Mannitol (Control), and after 1 day of severe NaCl or Mannitol treatments (300 mM). Data is shown as fold-changes relative to control conditions (i.e., all control values were set to 1, which is denoted by the dashed line). Primers used and gene information can be found in Supplementary Table 4. Values are means ± SE of 5–6 replicates and asterisks denote significant differences (*P* < 0.05) to the control condition for each gene expression analysis. Letters denote significant differences (*P* < 0.05) among genotypes and stress conditions for each gene expression analysis.

For the statistical analyses in Figures 1–4 and 6, a one-way ANOVA with a level of significance of *P* < 0.05 was performed with SPSS statistical software package, version 25 (IBM Corp., 2016, Armonk, New York, NY, USA), and Duncan’s posthoc test was used to determine statistically significant differences. In addition, Student’s t tests were used for the statistical analyses in Figures 1B, 5, 6, Supplementary Figure 1, Supplementary Tables 2,3 in order to determine significant (*P* < 0.05) differences between genotypes under each experimental condition (Figure 5, Supplementary Figure 1, Supplementary Tables 2,3), or to determine significant (*P* < 0.05) differences between control and stress treatments in each genotype (Figures 1B, 6, Supplementary Tables 2,3).

## Results

### The Alternative Oxidase Capacity and Expression in *aox1a* and Wild-Type Plants Under Control and Stress Conditions

The capacity of the alternative oxidase pathway (V_alt_), measured as the KCN-resistant respiration, was determined in both genotypes under control and stress treatments (Figure 1). As expected, V_alt_ was significantly (*P* < 0.05) lower in *aox1a* mutants than in WT plants (5.9 and 19.1 nmol O_2_ g^–1^ DW s^–1^, respectively) under control conditions. Under stress, V_alt_ increased in WT plants after salt treatment, while mannitol-treated plants displayed an intermediate V_alt_ (Figure 1A). On the other hand, V_alt_ remained similar in *aox1a* mutants under control and stress conditions, and it was much lower as compared to WT plants under stress conditions. Then, the AOX protein levels were determined in leaf crude extracts after immunoblotting analyses and quantified after normalization to mitochondrial porin levels (Figure 1B). In the line with respiratory capacity, the AOX protein levels were similarly increased after both stress treatments in WT plants. We did not detect any signal of AOX protein in the *aox1a* mutants as previously reported (Giraud et al., 2008; Strodtkötter et al., 2009). Thereafter, the expression profiles of the five genes encoding the AOX protein in *Arabidopsis* were determined by qPCR analyses in both genotypes under control and stress conditions (Figure 1C). Transcript levels of the *AOX1a* gene were not detected in the *aox1a* mutant, thus confirming the expected effect of the *aox1a* mutation, while the expression of this gene was induced in WT plants under both stress conditions (Figure 1C), particularly under salinity (7.2-fold increase). The transcript levels of *AOX1b* and *AOX2* were below the level of detection in all control and most of the stress-treated samples. The transcript levels of *AOX1c* were generally not responsive to stress, except for the slight significant (*P* < 0.05) increase in WT plants after mannitol treatment. On the other hand, the levels of *AOX1d* transcripts were highly responsive to both stress conditions, particularly in the *aox1a* mutants, which display a much higher response than in WT plants.

### Leaf Phenotype and Water Status in *aox1a* and Wild-Type Plants Under Control and Stress Conditions

Wild-type (WT) and *aox1a* mutant plants did not show visual differences under control conditions, as previously observed in several other studies (Figure 2A). Acute salt and mannitol treatments caused minor leaf wilting in both WT and *aox1a* mutant plants after one day of stress (Figure 2A). Leaf relative water content (RWC) was determined as an indicator of water status in both genotypes after salt and mannitol treatments (Figure 2B). Salinity provoked a reduction of leaf RWC in both genotypes, while a significant (*P* < 0.05) reduction of leaf RWC after mannitol treatment was only observed in the *aox1a* mutants. Under both stress conditions, the leaf RWC was significantly (*P* < 0.05) lower in the *aox1a* mutants as compared to WT plants.

### Respiration and Electron Partitioning to the Alternative Oxidase Pathway in *aox1a* and Wild-Type Plants Under Control and Stress Conditions

Both total respiration and the electron partitioning between the cytochrome oxidase pathway (COP) and the AOP were determined by using the oxygen isotope discrimination technique (Figure 3). Total oxygen uptake (V_t_) and electron partitioning to the alternative pathway (τ_a_) were similar in WT and *aox1a* mutants under control conditions. Salt and mannitol treatments significantly (*P* < 0.05) increased respiration only in WT plants from 23.9 to 36.6 and 32.9 nmol O_2_ g^–1^ DW s^–1^, respectively. Similarly, τ_a_ significantly increased after salt and mannitol treatments in WT plants from 0.17 to 0.33 and 0.28, respectively. On the other hand, no significant (*P* < 0.05) change in τ_a_ was observed in *aox1a* mutants after salt and mannitol treatments. The *in vivo* AOP activity (ν_alt_) was similar in both lines under control conditions and increased significantly in WT plants after salt (1.94-fold) and mannitol (1.27-fold) treatments, whilst it was unaltered in *aox1a* mutants. The activity of the COP (ν_cyt_) was similar without significant (*P* < 0.05) differences between control and treated plants in both genotypes.

### Photosynthetic Parameters in *aox1a* and Wild-Type Plants Under Control and Stress Conditions

The chloroplast electron transport rate (ETR) was determined at different light intensities in *aox1a* and WT plants under control and stress conditions (Figure 4A). While similar light-response curves were obtained in both genotypes under control conditions, some differences were observed under stress (Figure 4A). Light curves were modelled to obtain photosynthetic parameters (see section “Materials and Methods” for details) such as alpha, which indicates the photosynthetic rate in the light-limited region of the light curve, and the maximum electron transport rate (ETR_max_; Figure 4B). Both parameters were similar among genotypes under control conditions. Regarding alpha, only *aox1a* mutants displayed a significant (*P* < 0.05) reduction after salt treatment as compared to control conditions, and this parameter became significantly (*P* < 0.05) lower in *aox1a* as compared to WT under salinity. On the other hand, the ETR_max_ was clearly reduced under both stress conditions and in both genotypes but notably, this parameter was significantly lower in the *aox1a* mutants as compared to WT plants under mannitol stress. In addition, the maximum efficiency of PSII (F_v_/F_m_) was determined by measuring chlorophyll fluorescence after dark adaptation, in order to evaluate the level of photoinhibitory stress after salt and mannitol treatments (Figure 4B). The F_v_/F_m_ was also reduced in both genotypes under both stress conditions, and salinity was the condition inducing higher photoinhibition in both genotypes (Figure 4B). Notably, the *aox1a* mutants displayed significantly lower F_v_/F_m_ after both stress treatments as compared to WT plants (Figure 4B). Overall, these results indicate a similar photosynthetic performance among genotypes under control conditions, however, the *aox1a* mutants displayed higher photoinhibition and lower photosynthetic activity under stress conditions.

### Metabolite Profiling in *aox1a* and Wild-Type Plants Under Control and Stress Conditions

In order to further investigate the metabolic changes underlying the different physiological responses observed in the *aox1a* mutants, gas chromatography-time of flight-mass spectrometry (GC–TOF–MS) metabolite profiling analysis was performed on rosette leaves from plants grown under control and stress conditions (Figure 5 and Supplementary Table 2). A total of 45 metabolites were identified using gas chromatography-mass spectrometry (GC-MS), including several amino acids, organic acids, sugars and sugar alcohols (Supplementary Tables 1,2; Figure 5). Relative metabolite levels were normalized to the mean levels of WT plants under control conditions (Supplementary Table 1 and Figure 5). Under control conditions, the *aox1a* mutants displayed higher levels of putrescine (2.31-fold), glycine (1.37-fold), erythritol (2.59-fold) and maltose (1.69-fold) as well as of the TCA cycle intermediates, citrate (1.62-fold), fumarate (1.30-fold) and malate (1.55-fold). Under stress, leaves of WT plants displayed significant increases in 31 metabolites after salt and mannitol treatments (Figure 5 and Supplementary Table 2). While many of the metabolites increasing after mannitol treatment were the same as those increasing under salinity, there were several exceptions: alanine, glutamate, ornithine, citrate, glycerol and urea, which did not significantly (*P* < 0.05) increase after mannitol treatment; and glutamine, fumarate, erythritol, galactinol, xylose and trehalose, which increased only after mannitol treatment (Figure 5 and Supplementary Table 2). On the other hand, only glycerate and pyruvate displayed decreased levels after both stress treatments, and dehydroascorbate was reduced only after salt treatment (Figure 5 and Supplementary Table 2).

Metabolite levels in the *aox1a* mutants displayed similar patterns after both stress treatments as compared with WT plants (Figure 5). Nevertheless, the levels of asparagine, glycine, proline, malate and spermidine, although following similar stress-induced patterns in both genotypes, were significantly (*P* < 0.05) higher in *aox1a* mutants than in WT plants under salinity (Figure 5). On the other hand, aspartate, dehydroascorbate and fructose were significantly (*P* < 0.05) lower in *aox1a* mutants than in WT plants after salt treatment. Regarding mannitol stress, only aspartate and glycerate levels were significantly (*P* < 0.05) higher in *aox1a* mutants than in WT plants. On the other hand, fumarate, citrate, fructose and glucose were significantly (*P* < 0.05) lower in *aox1a* mutants than in WT plants after mannitol treatment.

Additionally, absolute levels of metabolites were determined to evaluate the physiological relevance for the accumulation of the stress-related metabolites with protective functions. The absolute levels of 37 metabolites, present in our standard mixture, were determined for all conditions in both genotypes (Supplementary Figure 2 and Supplementary Table 3). Metabolites in order of absolute abundance under salinity conditions are shown in Supplementary Figure 3. Among the metabolites observed as significantly different between WT or *aox1a* mutants under salinity (Figure 5 and Supplementary Table 2), glycine and proline were above 2 μmol gDW^–1^ in either WT or *aox1a* mutants, while malate, fructose and asparagine displayed lower amounts in the range of 0.3–0.8 μmol gDW^–1^ (Supplementary Figure 2 and Supplementary Table 3). With respect to mannitol treatment, glucose was by far the most abundant metabolite (56.5 and 34.1 μmol gDW^–1^ in WT and *aox1a* mutant, respectively) that was significantly lower in the *aox1a* mutants, followed by fumarate (10.4 and 7.6 μmol gDW^–1^) and fructose (4.1 and 2.0 μmol gDW^–1^; Supplementary Figure 2 and Supplementary Table 3).

### Gene Expression of Other Alternative Respiratory Components in *aox1a* and Wild-Type Plants Under Control and Stress Conditions

The relative levels of transcripts encoding mitochondrial proteins of the alternative respiratory pathways, other than AOX, were determined by qPCR in both WT and *aox1a* mutants (Supplementary Figure 1 and Figure 6). The aim of these gene expression analysis was to evaluate possible compensations from other alternative respiratory pathways for the lack of the *AOX1a* gene expression as well as the degree of mitochondrial stress perturbation. Regarding genes encoding alternative NAD(P)H dehydrogenases, transcript levels of *NDB3* were under the limit of detection while *NDB2* expression displayed a higher stress-response in the *aox1a* mutants as compared to WT (Figure 6), a similar trend as the observed for the *AOX1d* (Figure 1C). On the other hand, genes encoding *NDA1* and *NDA2* proteins were not responsive to stress conditions. Finally, the transcript levels of genes encoding uncoupling proteins (UCPs) were also evaluated (Supplementary Figure 1 and Figure 6); *UCP2* transcript levels were under the limit of detection while *UCP1* transcripts did not display any significant change under stress in both genotypes (Figure 6).

## Discussion

Responses of plant respiration to abiotic stresses are largely variable (Lambers and Oliveira, 2019; O’Leary et al., 2019). This variability can be partly explained by plants’ ability to adjust respiratory energy and carbon demands through changes in the partitioning of electrons between the cytochrome and alternative oxidase pathways (COP and AOP; Del-Saz et al., 2018; Lambers and Oliveira, 2019; O’Leary et al., 2019), without necessarily involve a major change in total respiration rate. In particular, the responses of the *in vivo* AOP and COP activities to salt (Martí et al., 2011; Del-Saz et al., 2016; Sánchez-Guerrero et al., 2019) and water stress (Guy and Vanlerberghe, 2005; Ribas-Carbo et al., 2005; Galle et al., 2010; Sanhueza et al., 2013) are variable, however, a common pattern emerge with the electron partitioning to AOX usually increasing, either by a decrease in COP activity, increase in AOP activity or both (Del-Saz et al., 2018). Notably, a specific increase in the *in vivo* AOP activity (i.e., in the absence of COP changes), was observed in leaves of *Medicago truncatula* genotypes after a sudden severe salt stress (Del-Saz et al., 2016). Such a particular AOP activation *in vivo* is in line with its proposed role in providing metabolic flexibility to carbon and energy metabolism under short-term environmental perturbations (Del-Saz et al., 2018), as well as its role as a first line of defence against oxidative stress (Møller, 2001). Following previous evidence, we have here investigated the role of the AOP under sudden acute salt stress in more detail. Our hypothesis was that *in vivo* AOP response in *aox1a* mutants will be curtailed under acute stress, which would impair stress-related metabolism as compared to WT plants. In addition, we also applied a high mannitol concentration to both genotypes for exploring whether similar effects on the respiration and stress-related metabolism were observed after an acute osmotic stress in the absence of ionic toxicity. We are aware that osmotic stress conditions applied here were not fully controlled by our experimental approach with plants grown in soil, however, our previous observations (Del-Saz et al., 2016) and those presented here show and track typical physiological (Figures 2,4) and molecular (Figure 5) responses to salt and water stress, thus allowing the establishment of relationships between the degree of water and (photo)oxidative stress with the metabolic changes studied.

### The Lack of *AOX1a* Does Not Impair Alternative Oxidase Pathway *in vivo* Activity Under Control Conditions but Restricted Its Response to Stress

As expected, the capacity of the AOP was significantly lower in the *aox1a* mutant than in WT plants (Figure 1A), although it was not completely abolished in agreement with previous observations (Watanabe et al., 2010; Vishwakarma et al., 2015; Zhang et al., 2016). In the present study, the oxygen isotope discrimination in the presence of KCN (Δ_a_ of approx. 31‰) provides additional evidence supporting that remaining O_2_ consumption after KCN treatment was mainly due to leaf AOX (maximum) activity. Indeed, *aox1a* mutants displayed similar total respiration, electron partitioning and *in vivo* AOP activity as WT plants under control conditions (Figure 3). These results are very similar to those reported in *AtAOX1a* anti-sense plants, previously obtained at the laboratory of the honourable Prof. James Siedow, grown under different light intensities (Florez-Sarasa et al., 2011). Our previous (Figure 1 from Florez-Sarasa et al., 2011) and current data (compare V_alt_ at Figure 1A with ν_alt_ at Figure 3) show that AOX is almost fully engaged (ν_alt_ to V_alt_ ratio close to 1) in *aox1a* impaired plants. Therefore, results presented here confirm that *AOX1a* isoform is not essential for maintaining leaf *in vivo* AOX activity under non-stress conditions. On the other hand, AOX protein signal could not be detected in the *aox1a* mutants under control conditions (Figure 1B) as in previous reports (Giraud et al., 2008; Strodtkötter et al., 2009; Oh et al., 2022). However, it is likely that samples after mitochondrial isolation or enrichment in mitochondrial membranes are required for the detection of low abundance AOX proteins (Oh et al., 2022). Indeed, AOX1D protein was detected in enriched mitochondrial membrane samples from *aox1a* mutants after proline incubation (Oh et al., 2022). Furthermore, evidence for the involvement in AOX capacity of isoforms other than AOX1a has recently been provided (Oh et al., 2022). To this respect, while single *aox1a* and *aox1d* mutants displayed lower AOX capacity as compared to WT plants, the reduction in AOX capacity was clearly more pronounced in double mutants *aox1a*-*aox1d* (Oh et al., 2022). While the relationship between protein levels and cyanide-resistant respiration may not be linear (Gonzalez-Meler et al., 1999) our and previous results (Oh et al., 2022) suggest that both AOX1a and AOX1d proteins can be responsible for AOX capacity in Arabidopsis plants. Notably, transcript levels of other AOX isoforms or alternative components of the mitochondrial respiratory chain in *aox1a* mutants were not higher than in WT plants (Supplementary Figure 1). Therefore, our and previous results in transcript and protein amounts suggest that *in vivo* AOP activity under non-stress conditions could be achieved without any additional induction of other AOX isoforms (i.e., AOX1c or AOX1d proteins). However, post-translational activation of the remaining AOX isoforms in *aox1a* mutants could also contribute to the observed *in vivo* AOP activity. To this respect, while moderate increases in the levels of some TCA cycle intermediates (citrate, malate and fumarate) were observed in *aox1a* mutants under control conditions (Figure 5), none of them have been shown to act as allosteric activators of any AOX isoform (Selinski et al., 2018b). However, analysis of AOX allosteric activators at the subcellular (mitochondrial) level would be required to attribute their effects on AOX activation *in vivo*.

Both stress treatments, significantly increased transcript levels of *AOX1a* and *AOX1d* in WT plants (Figure 1B), thus both isoforms could potentially contribute to the observed increase in AOP capacity (Figure 1A) and *in vivo* activity (Figure 3). On the other hand, the absence of *AOX1a* expression in the *aox1a* mutants coincided with a much higher induction of the *AOX1d* transcript levels (approx. 20 times higher) under both stress conditions, as compared to WT plants (Figure 1B). However, this response was not coupled to a significant (*P* < 0.05) increase in the AOX capacity or protein level under our experimental conditions (Figure 1). Moreover, the lack of *in vivo* AOP activity response observed in *aox1a* mutant leaves (Figure 3) clearly suggests that the absence of *AOX1a* was not compensated by other AOX isoforms (i.e., AOX1d) under stress conditions. These results are in line with previous observations suggesting no functional compensation for the lack of *AOX1a* under stress via high induction of *AOX1d* after respiratory inhibitor (Strodtkötter et al., 2009) and metal stress treatments (Keunen et al., 2015). In here, the *aox1a* mutants did not show respiratory response to acute salt and water stress, since AOP was the main contributor to this respiratory response in our experimental conditions (Figure 3). This AOP-specific response to acute salt stress was previously observed in a *Medicago truncatula* sensitive genotype (Del-Saz et al., 2016), which fits with the Arabidopsis behaviour, as a salt-sensitive species (Kazachkova et al., 2018). In addition, we here show that mannitol treatment induces a similar *in vivo* respiratory response as salt stress treatment (Figure 3), which suggests that the osmotic stress generated after both treatments is likely the main responsible for the observed *in vivo* AOP specific response. In fact, the lack of *in vivo* AOP response in *aox1a* mutants coincided with a more pronounced reduction in leaf RWC under both stress conditions, as compared to WT plants (Figure 2), which could be related to a reduced osmotic adjustment. Osmotic adjustment is an essential trait that plants use to maintain turgor by balancing the osmotic pressure imposed during salt or water stresses (Munns and Gilliham, 2015). While osmotic adjustment using inorganic ions is energetically “cheaper” than using organic solutes, salt-sensitive species usually rely in ion exclusion (particularly in leaves) and accumulation of organic solutes (Munns and Gilliham, 2015). In the present study, salt and mannitol treatments induced a large accumulation of several stress-related metabolites (SRMs; Figure 5), many of them being well-known for their roles in osmoregulation and osmoprotection (Slama et al., 2015).

### The Lack *in vivo* Alternative Oxidase Pathway Response Under Stress Altered the Accumulation of Stress-Related Metabolities Involved in Leaf Osmoregulation and Redox Balance

**FIGURE 7:**
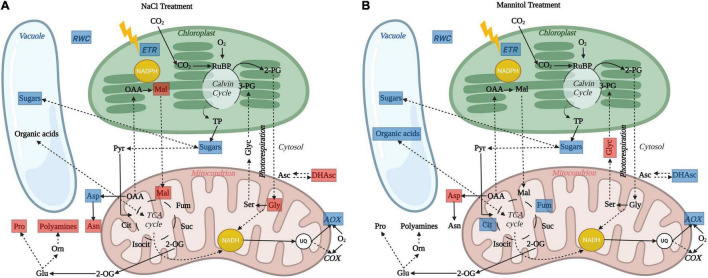
Schematic overview illustrating the impact of *in vivo* AOP restriction on leaf cell physiology and metabolism in *aox1a* plants after **(A)** salt and **(B)** mannitol treatments. Blue and red boxes indicate significant (*P* < 0.05) reductions and increases, respectively, in physiological indicators and metabolite levels in *aox1a* mutants as compared to WT plants. After both stress treatments, the lack of *in vivo* AOP response was associated to reduced sugars (glucose, fructose or both) accumulation in *aox1a* mutants, which can be partially explained by their lower photosynthetic activity. Reduced accumulation of sugars in *aox1a* mutants could affect their osmotic adjustment as reflected by their lower relative water content (RWC). The reduction of fumarate and citrate accumulation in *aox1a* plants, the former likely contributing the decreased osmotic adjustment, may be explained by a TCA activity impairment triggered by the lack of response on the *in vivo* AOP activity, which allows matrix NADH re-oxidation without energy demand (i.e., ATP for growth) constrains. This limitation in NADH reoxidation can also explain the accumulation of photorespiratory cycle intermediates glycine **(A)** and glycerate **(B)**. In addition, ion toxicity effects imposed by salinity could be aggravated by the lack of AOP response and, according to its role in avoiding ROS formation, thus trigger additional oxidative stress that was reflected in the alteration of other metabolic pathways involved in redox balancing and ROS detoxification **(A)**, as denoted by the accumulation of malate, dehydroascorbate, proline, polyamines (spermidine) and asparagine. Image created with BioRender (https://biorender.com).

Osmotic stress has immediate effects on plant growth, and the accumulation of osmolytes is part of the typical plant responses involved in osmotic adjustment (Munns and Gilliham, 2015). The accumulation of osmolytes is favoured under conditions when plant growth declines before photosynthesis, which can result in a relatively favourable carbon status allowing the use of photosynthates for osmolyte production at low cost (Hummel et al., 2010). In our short-term stress study, photosynthesis was not strongly affected, particularly at growth light intensity (Figure 4), which suggests minor limitations on C availability. In agreement, a large accumulation of sugars, sugar alcohols and amino acids was observed in both genotypes after stress treatments (Figure 5). Previous reports suggest that accumulation of sugars in leaves of Arabidopsis and other species after mannitol and salt treatments have a prominent role on osmotic adjustment (Thalmann and Santelia, 2017; Gurrieri et al., 2020). On the other hand, the accumulation of amino acids (i.e., proline) is a well-known mechanism contributing to osmoprotection and osmoregulation in different species (Slama et al., 2015). The relatively low concentrations of amino acids as typically observed in Arabidopsis leaves suggest their roles as osmoprotectants of subcellular structures, in scavenging reactive oxygen species or counteracting redox imbalances, rather than contributing to osmotic adjustment (Shabala and Shabala, 2011; Slama et al., 2015; Gurrieri et al., 2020). Our results are in line with these observations since the levels of sucrose, glucose and fructose were not only highly accumulated, but also reached the highest absolute amounts, as compared to highly accumulated amino acids such as proline and glycine (Supplementary Figure 2). Importantly, the accumulation of glucose and fructose in the *aox1a* mutants was lower as compared with WT plants (Figure 5), and together with their lower relative water content (RWC), suggests a decreased osmotic adjustment in the *aox1a* mutants (Figure 7). In this line, the *aox1a* mutants after mannitol treatment also displayed lower levels of fumarate, which was among the top three most abundant metabolites under stress (Supplementary Figure 2), and this metabolite was previously suggested to have a high contribution to osmotic adjustment in Arabidopsis under severe drought (Hummel et al., 2010). The lower photosynthetic performance of *aox1a* mutants could partly explain their lower sugar levels, which in turn could slow down the carbon flow through glycolysis and consequently the TCA cycle activity for the fumarate production (Figure 7). Nevertheless, fumarate can also be produced from malate which levels could be altered by the redox imbalances in *aox1a* plants. Furthermore, the accumulation of osmolytes is thought to be the result of the coordinated regulation of biosynthetic and catabolic pathways (Slama et al., 2015), many of which are tightly linked to respiratory pathways (Bandehagh and Taylor, 2020). Therefore, we cannot discard that the lower accumulation of fumarate and citrate observed in *aox1a* mutants after mannitol treatment could be partly due to a reduced TCA cycle activity as a direct consequence of a reduction in matrix NADH reoxidation by the AOP. Future experiments determining carbon fluxes (i.e., with ^13^C labelled substrates under control and osmotic stress conditions) could unveil the precise nature of the observed altered accumulation of TCA cycle intermediates.

Impairment of photosynthetic performance and higher photoinhibition have been observed in other *aox1a* suppressed plants under drought (Giraud et al., 2008; Dahal et al., 2014, 2017; Dahal and Vanlerberghe, 2017, 2018) high light (Florez-Sarasa et al., 2011) and temperature (Watanabe et al., 2010; Li et al., 2020) stress, which have been attributed to the role of the AOP in providing flexibility to carbon and energy metabolism in photosynthetic tissues, particularly under stress (Del-Saz et al., 2018; Vanlerberghe et al., 2020). In our study, while both stress conditions induced photoinhibition in both genotypes (Figure 3), the decrease in Fv/Fm was higher under salinity as compared to mannitol stress (Figure 3), thus suggesting that ion toxicity probably led to higher oxidative stress at the chloroplast level. In line with a higher oxidative stress after salt treatment, as compared to mannitol treatment, both genotypes displayed higher levels of different metabolites with known functions on protecting against oxidative stress themselves (i.e., dehydroascorbate, proline, asparagine; Figure 5 and Supplementary Table 2) or as precursors of oxidative stress-related specialized metabolites (i.e., phenylalanine and tyrosine for phenylpropanoids synthesis; Figure 5 and Supplementary Table 2), as well as higher levels of transcripts encoding oxidative stress-responsive genes (*AOX1a* and *NDB2*; Figure 6). In agreement, the AOP activity in WT plants was significantly higher after salt than after mannitol stress (Figure 3). Importantly, the *aox1a* mutants, lacking stress response of the *in vivo* AOP activity, displayed higher levels of proline, asparagine, spemidine, and dehydroascorbate when compared to WT plants (Figures 5, 7). Increased levels of all these metabolites indicate a higher induction of metabolic pathways involved in redox balancing and avoidance of excessive ROS accumulation under stress (Szabados and Savouré, 2010; Maaroufi-Dguimi et al., 2011; Alcázar et al., 2020; Foyer et al., 2020). Moreover, higher glycine levels in *aox1a* mutants under salinity denotes a restriction in photorespiration due to reduction of glycine decarboxylation (Figure 7), as has been previously reported in *aox1a* mutants (Strodtkötter et al., 2009; Zhang et al., 2017; Li et al., 2020). Finally, higher malate levels in *aox1a* mutants could indicate an impairment in the reductant export from the chloroplast via the malate valve (Figure 7), as previously suggested (Yoshida et al., 2007; Dinakar et al., 2010; Vishwakarma et al., 2015).

## Conclusion

Mitochondrial function has been suggested to be essential for drought and salinity tolerance in plants as it can have roles in signal transduction, ion exclusion and homeostasis, ROS detoxification, the generation of ATP, and carbon balance (Bandehagh and Taylor, 2020; Munns et al., 2020; Vanlerberghe et al., 2020). However, information about the *in vivo* AOP and COP activity and their metabolic roles under water and salt stress remain scarce. The different metabolic responses in *aox1a* mutants lacking *in vivo* AOP response under stress (Figure 7) reinforce the relevant roles of the AOX previously suggested in redox balancing, avoidance of excessive ROS accumulation and photosynthetic performance. Furthermore, we here provide new evidence on the *in vivo* regulation and metabolic role of the AOP in the accumulation of stress-related metabolites associated to osmoregulation. While further experiments would be required to unravel the precise direction of subcellular carbon fluxes and its direct relationship with the *in vivo* AOP response, our results provide evidence on the contribution of the AOP in providing flexibility to carbon metabolism for biosynthesis, in addition to its role in cell redox balancing and preventing mitochondrial ROS formation (Smith et al., 2009; Vanlerberghe et al., 2020). Metabolites including TCA cycle intermediates (malate, citrate and fumarate), proline and hexoses, which were altered by the absence of *in vivo* AOP activity in this study, are similar to those typically altered when comparing salt-sensitive species *A. thaliana* with its relatives exhibiting salt-tolerance (Kazachkova et al., 2018). Therefore, our results provide important additional evidence reinforcing previous suggestions for AOX manipulation as a reasonable strategy for improving salt-tolerance in salt-sensitive crops (Smith et al., 2009), notwithstanding other important strategies involving salt-tolerant crops (Ventura et al., 2015; Fernie and Yan, 2019). Whether a higher AOP capacity or activity is primed for a better performance in case of salinity exposure remains to be investigated in a wider range of salt-tolerant crops. In addition, the relevance of AOP and its coordinated regulation with other alternative respiratory pathways under longer exposure to salt and/or water stress remains to be investigated in depth.

## Data Availability Statement

The original contributions presented in the study are included in the article/Supplementary Material, further inquiries can be directed to the corresponding authors.

## Author Contributions

ND-S, MR-C, and IF-S conceived and designed the idea of this experiment. ND-S, AI-S, and DA-F carried out oxygen electrode and mass spectrometry measurements of respiration as well as leaf relative water content determinations. AI-S performed photosynthetic and gene expression analyses. ML-G, FP, and MC-M performed samples preparation, metabolite extractions and analyses. IF-S and AF performed the metabolite data analysis. IF-S and ND-S performed the statistical analysis. ND-S and IF-S wrote the first draft of the manuscript with subsequent inputs from all co-authors. All authors have read and agreed to the published version of the manuscript.

## Conflict of Interest

The authors declare that the research was conducted in the absence of any commercial or financial relationships that could be construed as a potential conflict of interest.

## Publisher’s Note

All claims expressed in this article are solely those of the authors and do not necessarily represent those of their affiliated organizations, or those of the publisher, the editors and the reviewers. Any product that may be evaluated in this article, or claim that may be made by its manufacturer, is not guaranteed or endorsed by the publisher.

## Footnotes

1
http://gmd.mpimp-golm.mpg.de/

2
http://www.nanodrop.com/

## Supplementary Material

Supplementary Figure 1 Gene expression analyses of alternative respiratory components under control conditions. Transcript levels of genes encoding alternative oxidases, uncoupling proteins and alternative NAD(P)H dehydrogenases were determined by qPCR analyses (see Materials and Methods for details) in leaves of wild-type Col 0 (WT) and *aox1a A. thaliana* plants as described in Figure 5A, 6. Data is shown as fold-changes relative to WT (i.e., all WT values were set to 1, which is denoted by the dashed line). Primers used and gene information can be found in Supplementary Table 4. Values are means ± SE of 5–6 replicates and asterisks denote significant differences (*P* < 0.05) to the WT for each gene expression analysis. n.d. (not detected).

Supplementary Figure 2 Metabolite profiles showing absolute changes in primary metabolism. Coloured table showing the absolute levels (μmol gDW^–1^) of the GC-MS-analysed metabolites in leaves of wild-type Col 0 (WT) and *aox1a A. thaliana* plants untreated with NaCl or Mannitol (Control), and after 1 day of severe NaCl or Mannitol treatments (300 mM). Metabolites are shown in order of absolute abundance under 300 mM NaCl conditions. In this table, the intensity of the red colour indicates the absolute level of the metabolite as well as the green bars inside the cells. Values are means of 4–6 replicates and the statistical differences to the WT plants in each experimental condition as well as between control and stress treatments in each genotype are presented in Supplementary Table 3.

## References

[B1] Alcázar R. Bueno M. Tiburcio A. F. (2020). Polyamines: small amines with large effects on plant abiotic stress tolerance. *Cells* 9:2373. 10.3390/cells9112373 33138071 PMC7692116

[B2] Bandehagh A. Taylor N. L. (2020). Can alternative metabolic pathways and shunts overcome salinity induced inhibition of central carbon metabolism in crops? *Front. Plant Sci.* 11:1072. 10.3389/fpls.2020.01072 32849676 PMC7417600

[B3] Batista-Silva W. Heinemann B. Rugen N. Nunes-Nesi A. Araújo W. L. Braun H. P. (2019). The role of amino acid metabolism during abiotic stress release. *Plant Cell Environ.* 42 1630–1644. 10.1111/pce.13518 30632176

[B4] Clifton R. Millar A. H. Whelan J. (2006). Alternative oxidases in *Arabidopsis*: a comparative analysis of differential expression in the gene family provides new insights into function of non-phosphorylating bypasses. *Biochim. Biophys. Acta Bioenerget.* 1757 730–741. 10.1016/j.bbabio.2006.03.009 16859634

[B5] Dahal K. Martyn G. D. Alber N. A. Vanlerberghe G. C. (2017). Coordinated regulation of photosynthetic and respiratory components is necessary to maintain chloroplast energy balance in varied growth conditions. *J. Exp. Bot.* 68 657–671. 10.1093/jxb/erw469 28011719 PMC5441918

[B6] Dahal K. Vanlerberghe G. C. (2017). Alternative oxidase respiration maintains both mitochondrial and chloroplast function during drought. *New Phytol.* 213 560–571. 10.1111/nph.14169 27579773

[B7] Dahal K. Vanlerberghe G. C. (2018). Improved chloroplast energy balance during water deficit enhances plant growth: more crop per drop. *J. Exp. Bot.* 69 1183–1197. 10.1093/jxb/erx474 29281082 PMC6018952

[B8] Dahal K. Wang J. Martyn G. D. Rahimy F. Vanlerberghe G. C. (2014). Mitochondrial alternative oxidasemaintains respiration and preserves photosynthetic capacity during moderate drought in *Nicotiana tabacum*. *Plant Physiol.* 166 1560–1574. 10.1104/pp.114.247866 25204647 PMC4226348

[B9] Darko E. Végh B. Khalil R. Marček T. Szalai G. Pál M. (2019). Metabolic responses of wheat seedlings to osmotic stress induced by various osmolytes under iso-osmotic conditions. *PLoS One* 14:e0226151. 10.1371/journal.pone.0226151 31856179 PMC6922385

[B10] Day D. A. Krab K. Lambers H. Moore A. L. Siedow J. N. Wagner A. M. (1996). The cyanide-resistant ocidase: to inhibit or not to inhibit, that is the question. *Plant Physiol.* 110 1–2. 10.1104/pp.110.1.1 12226168 PMC157687

[B11] Del-Saz N. F. Florez-Sarasa I. Clemente-Moreno M. J. Mhadhbi H. Flexas J. Fernie A. R. (2016). Salinity tolerance is related to cyanide-resistant alternative respiration in *Medicago truncatula* under sudden severe stress. *Plant Cell Environ.* 39 2361–2369. 10.1111/pce.12776 27304415

[B12] Del-Saz N. F. Ribas-Carbo M. Martorell G. Fernie A. R. Florez-Sarasa I. (2017). “Measurements of electron partitioning between cytochrome and alternative oxidase pathways in plant tissues,” in *Methods in Molecular Biology*, ed. Jagadis Gupta K. (New York, NY: Humana Press Inc), 203–217. 10.1007/978-1-4939-7292-0_17 28871545

[B13] Del-Saz N. F. Ribas-Carbo M. McDonald A. E. Lambers H. Fernie A. R. Florez-Sarasa I. (2018). An in vivo perspective of the role(s) of the alternative oxidase pathway. *Trends Plant Sci.* 23 206–219. 10.1016/j.tplants.2017.11.006 29269217

[B14] Dinakar C. Raghavendra A. S. Padmasree K. (2010). Importance of AOX pathway in optimizing photosynthesis under high light stress: role of pyruvate and malate in activating AOX. *Physiol. Plant.* 139 13–26. 10.1111/j.1399-3054.2010.01346.x 20059739

[B15] Epstein E. H. Jr. Levin D. L. Croft J. D. Jr. Lutzner M. A. (1972). MYCOSIS FUNGOIDES. *Medicine* 51 61–72. 5009530

[B16] Fàbregas N. Fernie A. R. (2019). The metabolic response to drought. *J. Exp. Bot.* 70 1077–1085. 10.1093/jxb/ery437 30726961

[B17] Fernie A. R. Aharoni A. Willmitzer L. Stitt M. Tohge T. Kopka J. (2011). Recommendations for reporting metabolite data. *Plant Cell* 23 2477–2482. 10.1105/tpc.111.086272 21771932 PMC3226225

[B18] Fernie A. R. Yan J. (2019). De novo domestication: an alternative route toward new crops for the future. *Mol. Plant* 12 615–631. 10.1016/j.molp.2019.03.016 30999078

[B19] Florez-Sarasa I. Flexas J. Rasmusson A. G. Umbach A. L. Siedow J. N. Ribas-Carbo M. (2011). In vivo cytochrome and alternative pathway respiration in leaves of *Arabidopsis thaliana* plants with altered alternative oxidase under different light conditions. *Plant, Cell Environ.* 34 1373–1383. 10.1111/j.1365-3040.2011.02337.x 21486306

[B20] Florez-Sarasa I. Noguchi K. Araújo W. L. Garcia-Nogales A. Fernie A. R. Flexas J. (2016). Impaired cyclic electron flow around photosystem I disturbs high-light respiratory metabolism. *Plant Physiol.* 172 2176–2189. 10.1104/pp.16.01025 27760881 PMC5129710

[B21] Foyer C. H. Kyndt T. Hancock R. D. (2020). Vitamin C in plants: novel concepts, new perspectives, and outstanding issues. *Antioxidants Redox Signal.* 32 463–485. 10.1089/ars.2019.7819 31701753

[B22] Galle A. Florez-Sarasa I. Thameur A. de Paepe R. Flexas J. Ribas-Carbo M. (2010). Effects of drought stress and subsequent rewatering on photosynthetic and respiratory pathways in *Nicotiana sylvestris* wild type and the mitochondrial complex I-deficient CMSII mutant. *J. Exp. Bot.* 61 765–775. 10.1093/jxb/erp344 19933320 PMC2814110

[B23] Garmash E. V. Velegzhaninov I. O. Ermolina K. V. Rybak A. V. Malyshev R. V. (2020). Altered levels of AOX1a expression result in changes in metabolic pathways in *Arabidopsis thaliana* plants acclimated to low dose rates of ultraviolet B radiation. *Plant Sci.* 291:110332. 10.1016/j.plantsci.2019.110332 31928662

[B24] Giraud E. Ho L. H. M. Clifton R. Carroll A. Estavillo G. Tan Y. F. (2008). The absence of Alternative Oxidase1a in *Arabidopsis* results in acute sensitivity to combined light and drought stress. *Plant Physiol.* 147 595–610. 10.1104/pp.107.115121 18424626 PMC2409015

[B25] Gong Q. Li S. Zheng Y. Duan H. Xiao F. Zhuang Y. (2020). SUMOylation of MYB30 enhances salt tolerance by elevating alternative respiration via transcriptionally upregulating AOX1a in *Arabidopsis*. *Plant J.* 102 1157–1171. 10.1111/tpj.14689 31951058

[B26] Gonzalez-Meler M. A. Ribas-Carbo M. Giles L. Siedow J. N. (1999). The effect of growth and measurement temperature on the activity of the alternative respiratory pathway. *Plant Physiol.* 120 765–772. 10.1104/pp.120.3.765 10398711 PMC59314

[B27] Gurrieri L. Merico M. Trost P. Forlani G. Sparla F. (2020). Impact of drought on soluble sugars and free proline content in selected *Arabidopsis mutants*. *Biology (Basel)* 9 1–14. 10.3390/biology9110367 33137965 PMC7692697

[B28] Guy R. D. Vanlerberghe G. C. (2005). Partitioning of respiratory electrons in the dark in leaves of transgenic tobacco with modified levels of alternative oxidase. *Physiol. Plant.* 125 171–180. 10.1111/j.1399-3054.2005.00557.x

[B29] Hummel I. Pantin F. Sulpice R. Piques M. Rolland G. Dauzat M. (2010). *Arabidopsis* plants acclimate to water deficit at low cost through changes of carbon usage: an integrated perspective using growth, metabolite, enzyme, and gene expression analysis. *Plant Physiol.* 154 357–372. 10.1104/pp.110.157008 20631317 PMC2938159

[B30] Kazachkova Y. Eshel G. Pantha P. Cheeseman J. M. Dassanayake M. Barak S. (2018). Halophytism: what have we learnt from *Arabidopsis thaliana* relative model systems? *Plant Physiol.* 178 972–988. 10.1104/pp.18.00863 30237204 PMC6236594

[B31] Keunen E. Schellingen K. van der Straeten D. Remans T. Colpaert J. Vangronsveld J. (2015). ALTERNATIVE OXIDASE1a modulates the oxidative challenge during moderate Cd exposure in *Arabidopsis thaliana* leaves. *J. Exp. Bot.* 66 2967–2977. 10.1093/jxb/erv035 25743159

[B32] Kopka J. Schauer N. Krueger S. Birkemeyer C. Usadel B. Bergmüller E. (2005). GMD@CSB.DB: the Golm metabolome database. *Bioinformatics* 21 1635–1638. 10.1093/bioinformatics/bti236 15613389

[B33] Lambers H. Oliveira R. S. (2019). “Photosynthesis, respiration, and long-distance transport: photosynthesis,” in *Plant Physiological Ecology*, eds Lambers H. Oliveira R. S. (Cham: Springer), 11–114. 10.1007/978-3-030-29639-1_2

[B34] Lisec J. Schauer N. Kopka J. Willmitzer L. Fernie A. R. (2006). Gas chromatography mass spectrometry-based metabolite profiling in plants. *Nat. Protoc.* 1 387–396. 10.1038/nprot.2006.59 17406261

[B35] Livak K. J. Schmittgen T. D. (2001). Analysis of relative gene expression data using real-time quantitative PCR and the 2-ΔΔCT method. *Methods* 25 402–408. 10.1006/meth.2001.1262 11846609

[B36] Li Y. T. Liu M. J. Li Y. Liu P. Zhao S. J. Gao H. Y. (2020). Photoprotection by mitochondrial alternative pathway is enhanced at heat but disabled at chilling. *Plant J.* 104 403–415. 10.1111/tpj.14931 32683757

[B37] Luedemann A. von Malotky L. Erban A. Kopka J. (2012). TagFinder: preprocessing software for the fingerprinting and the profiling of gas chromatography-mass spectrometry based metabolome analyses. *Methods Mol. Biol.* 860 255–286. 10.1007/978-1-61779-594-7_16 22351182

[B38] Maaroufi-Dguimi H. Debouba M. Gaufichon L. Clément G. Gouia H. Hajjaji A. (2011). An *Arabidopsis mutant* disrupted in ASN2 encoding asparagine synthetase 2 exhibits low salt stress tolerance. *Plant Physiol. Biochem.* 49 623–628. 10.1016/j.plaphy.2011.03.010 21478030

[B39] Martí M. C. Florez-Sarasa I. Camejo D. Ribas-Carbó M. Lázaro J. J. Sevilla F. (2011). Response of mitochondrial thioredoxin PsTrxo1, antioxidant enzymes, and respiration to salinity in pea (*Pisum sativum* L.) leaves. *J. Exp. Bot.* 62 3863–3874. 10.1093/jxb/err076 21460385 PMC3134343

[B40] Mattoo A. K. Sobolev A. P. Neelam A. Goyal R. K. Handa A. K. Segre A. L. (2006). Nuclear magnetic resonance spectroscopy-based metabolite profiling of transgenic tomato fruit engineered to accumulate spermidine and spermine reveals enhanced anabolic and nitrogen-carbon interactions. *Plant Physiol.* 142 1759–1770. 10.1104/pp.106.084400 17041034 PMC1676058

[B41] Møller I. M. (2001). Plant mitochondria and oxidative stress: electron transport, nadph turnover, and metabolism of reactive Oxygen Species. *Annu. Rev. Plant Physiol. Plant Mol. Biol.* 52 561–591. 10.1146/annurev.arplant.52.1.561 11337409

[B42] Morelli L. Paulišić S. Qin W. Iglesias-Sanchez A. Roig-Villanova I. Florez-Sarasa I. (2021). Light signals generated by vegetation shade facilitate acclimation to low light in shade-avoider plants. *Plant Physiol.* 186 2137–2151. 10.1093/plphys/kiab206 34618102 PMC8331150

[B43] Munns R. Day D. A. Fricke W. Watt M. Arsova B. Barkla B. J. (2020). Energy costs of salt tolerance in crop plants. *New Phytol.* 225 1072–1090. 10.1111/nph.15864 31004496

[B44] Munns R. Gilliham M. (2015). Salinity tolerance of crops – what is the cost? *New Phytol.* 208 668–673. 10.1111/nph.13519 26108441

[B45] Oh G. G. K. O’Leary B. M. Signorelli S. Millar A. H. (2022). Alternative oxidase (AOX) 1a and 1d limit proline-induced oxidative stress and aid salinity recovery in *Arabidopsis*. *Plant Physiol.* 188 1521–1536. 10.1093/plphys/kiab578 34919733 PMC8896607

[B46] O’Leary B. M. Asao S. Millar A. H. Atkin O. K. (2019). Core principles which explain variation in respiration across biological scales. *New Phytol.* 222 670–686. 10.1111/nph.15576 30394553

[B47] Pires M. V. Pereira Júnior A. A. Medeiros D. B. Daloso D. M. Pham P. A. Barros K. A. (2016). The influence of alternative pathways of respiration that utilize branched-chain amino acids following water shortage in *Arabidopsis*. *Plant Cell Environ.* 39 1304–1319. 10.1111/pce.12682 26616144

[B48] Platt T. Gallegos C. L. (1980). “Modelling primary production,” in *Primary Productivity in the Sea*, ed. Falkowski P. G. (Boston, MA: Springer), 339–362. 10.1007/978-1-4684-3890-1_19

[B49] Rasmusson A. G. Fernie A. R. van Dongen J. T. (2009). Alternative oxidase: a defence against metabolic fluctuations? *Physiol. Plant.* 137 371–382. 10.1111/j.1399-3054.2009.01252.x 19558416

[B50] Ribas-Carbo M. Taylor N. L. Giles L. Busquets S. Finnegan P. M. Day D. A. (2005). Effects of water stress on respiration in soybean leaves. *Plant Physiol.* 139 466–473. 10.1104/pp.105.065565 16126857 PMC1203395

[B51] Saisho D. Nambara E. Naito S. Tsutsumi N. Hirai A. Nakazono M. (1997). *Characterization of the Gene Family for Alternative Oxidase From Arabidopsis thaliana.* Alphen aan den Rijn: Kluwer Academic Publishers, 10.1023/A:1005818507743 9349280

[B52] Sanchez D. H. Siahpoosh M. R. Roessner U. Udvardi M. Kopka J. (2008). Plant metabolomics reveals conserved and divergent metabolic responses to salinity. *Physiol. Plant.* 132 209–219. 10.1111/j.1399-3054.2007.00993.x 18251862

[B53] Sánchez-Guerrero A. Fernández del-Saz N. Florez-Sarasa I. Ribas-Carbó M. Fernie A. R. Jiménez A. (2019). Coordinated responses of mitochondrial antioxidative enzymes, respiratory pathways and metabolism in *Arabidopsis thaliana* thioredoxin trxo1 mutants under salinity. *Environ. Exp. Bot.* 162 212–222. 10.1016/j.envexpbot.2019.02.026

[B54] Sanhueza C. Bascunan-Godoy L. Corcuera L. J. Turnbull M. H. (2013). The response of leaf respiration to water stress in *Nothofagus* species. *N. Z. J. Bot.* 51 88–103. 10.1080/0028825X.2012.759600

[B55] Selinski J. Scheibe R. Day D. A. Whelan J. (2018a). Alternative oxidase is positive for plant performance. *Trends Plant Sci.* 23 588–597. 10.1016/j.tplants.2018.03.012 29665989

[B56] Selinski J. Hartmann A. Deckers-Hebestreit G. Day D. A. Whelan J. Scheibe R. (2018b). Alternative oxidase isoforms are differentially activated by tricarboxylic acid cycle intermediates. *Plant Physiol.* 176 1423–1432. 10.1104/pp.17.01331 29208641 PMC5813554

[B57] Shabala S. Shabala L. (2011). Ion transport and osmotic adjustment in plants and bacteria. *Biomol. Concepts* 2 407–419. 10.1515/BMC.2011.032 25962045

[B58] Slama I. Abdelly C. Bouchereau A. Flowers T. Savouré A. (2015). Diversity, distribution and roles of osmoprotective compounds accumulated in halophytes under abiotic stress. *Ann. Bot.* 115 433–447. 10.1093/aob/mcu239 25564467 PMC4332610

[B59] Smith C. A. Melino V. J. Sweetman C. Soole K. L. (2009). Manipulation of alternative oxidase can influence salt tolerance in *Arabidopsis thaliana*. *Physiol. Plant.* 137 459–472. 10.1111/j.1399-3054.2009.01305.x 19941623

[B60] Strodtkötter I. Padmasree K. Dinakar C. Speth B. Niazi P. S. Wojtera J. (2009). Induction of the AOX1D isoform of alternative oxidase in *A. thaliana* T-DNA insertion lines lacking isoform AOX1A is insufficient to optimize photosynthesis when treated with antimycin a. *Mol. Plant* 2 284–297. 10.1093/mp/ssn089 19825614

[B61] Szabados L. Savouré A. (2010). Proline: a multifunctional amino acid. *Trends Plant Sci.* 15 89–97. 10.1016/j.tplants.2009.11.009 20036181

[B62] Thalmann M. Santelia D. (2017). Starch as a determinant of plant fitness under abiotic stress. *New Phytol.* 214 943–951. 10.1111/nph.14491 28277621

[B63] Umbach A. L. Fiorani F. Siedow J. N. (2005). Characterization of transformed *Arabidopsis* with altered alternative oxidase levels and analysis of effects on reactive oxygen species in tissue. *Plant Physiol.* 139 1806–1820. 10.1104/pp.105.070763 16299171 PMC1310561

[B64] Vanlerberghe G. C. (2013). Alternative oxidase: a mitochondrial respiratory pathway to maintain metabolic and signaling homeostasis during abiotic and biotic stress in plants. *Int. J. Mol. Sci.* 14 6805–6847. 10.3390/ijms14046805 23531539 PMC3645666

[B65] Vanlerberghe G. C. Dahal K. Alber N. A. Chadee A. (2020). Photosynthesis, respiration and growth: a carbon and energy balancing act for alternative oxidase. *Mitochondrion* 52 197–211. 10.1016/j.mito.2020.04.001 32278748

[B66] Ventura Y. Eshel A. Pasternak D. Sagi M. (2015). The development of halophyte-based agriculture: past and present. *Ann. Bot.* 115 529–540. 10.1093/aob/mcu173 25122652 PMC4332600

[B67] Vishwakarma A. Tetali S. D. Selinski J. Scheibe R. Padmasree K. (2015). Importance of the alternative oxidase (AOX) pathway in regulating cellular redox and ROS homeostasis to optimize photosynthesis during restriction of the cytochrome oxidase pathway in *Arabidopsis thaliana*. *Ann. Bot.* 116 555–569. 10.1093/aob/mcv122 26292995 PMC4578005

[B68] Watanabe C. K. Hachiya T. Takahara K. Kawai-Yamada M. Uchimiya H. Uesono Y. (2010). Effects of aox1a deficiency on plant growth, gene expression of respiratory components and metabolic profile under low-nitrogen stress in *Arabidopsis thaliana*. *Plant Cell Physiol.* 51 810–822. 10.1093/pcp/pcq033 20304787

[B69] Xu F. Yuan S. Zhang D. W. Lv X. Lin H. H. (2012). The role of alternative oxidase in tomato fruit ripening and its regulatory interaction with ethylene. *J. Exp. Bot.* 63 5705–5716. 10.1093/jxb/ers226 22915749 PMC3444281

[B70] Yoshida K. Terashima I. Noguchi K. (2007). Up-regulation of mitochondrial alternative oxidase concomitant with chloroplast over-reduction by excess light. *Plant Cell Physiol.* 48 606–614. 10.1093/pcp/pcm033 17339232

[B71] Zhang D. W. Yuan S. Xu F. Zhu F. Yuan M. Ye H. X. (2016). Light intensity affects chlorophyll synthesis during greening process by metabolite signal from mitochondrial alternative oxidase in *Arabidopsis*. *Plant Cell Environ.* 39 12–25. 10.1111/pce.12438 25158995

[B72] Zhang Z. S. Liu M. J. Scheibe R. Selinski J. Zhang L. T. Yang C. (2017). Contribution of the alternative respiratory pathway to PSII photoprotection in C3 and C4 plants. *Mol. Plant* 10 131–142. 10.1016/j.molp.2016.10.004 27746301

[B73] Zhu T. Zou L. Li Y. Yao X. Xu F. Deng X. (2018). Mitochondrial alternative oxidase-dependent autophagy involved in ethylene-mediated drought tolerance in *Solanum lycopersicum*. *Plant Biotechnol. J.* 16 2063–2076. 10.1111/pbi.12939 29729068 PMC6230944

