# Supplementary material for: The Lack of Alternative Oxidase 1a Restricts in vivo Respiratory Activity and Stress-Related Metabolism for Leaf Osmoprotection and Redox Balancing Under Sudden Acute Water and Salt Stress in Arabidopsis thaliana
Source: Front Plant Sci. 2022 May 17;13:833113. doi: 10.3389/fpls.2022.833113 (PMC9152546; doi:10.3389/fpls.2022.833113)
Supplement: Supplementary file 2 [file Table_2.DOCX]

**Supplemental Table 2.** Relative metabolite levels in leaves of WT and *aox1a* plants under control conditions and after 1 day of severe (300 mM) NaCl and Mannitol treatments as measured by GC-MS (see material and methods for details). Data is presented as means ± SE for 4 to 6 biological replicates normalized to the mean level of the WT plants under control conditions. Bold numbers denote significant differences (*P* < 0.05) to the control condition in each genotype separately. Asterisks denote significant differences (*P* < 0.05) between WT and *aox1a* plants in each experimental condition. †Denotes metabolites detected only in one replicate in WT at control conditions. ‘n.d.’ denotes cases for not detected metabolites.

|  | **Control** | |  | | **NaCl** | |  | | **Mannitol** | |  |  |
| --- | --- | --- | --- | --- | --- | --- | --- | --- | --- | --- | --- | --- |
| Metabolite | *WT* | *AOX1a* |  |  | *WT* | *AOX1a* |  |  | *WT* | *AOX1a* | |  |
| **Amino acids** |  |  |  |  |  |  |  |  |  |  | |  |
| Alanine | 1±0.29 | 0.94±0.23 |  |  | **1.47±0.13** | 1.71±0.27 |  |  | 1.80±0.43 | **2.17±0.30** | |  |
| Asparagine | 1±0.15 | 0.90±0.06 |  |  | **3.24±0.25** | **4.18±0.25*** |  |  | **2.07±0.35** | 1.97±0.47 | |  |
| Aspartate | 1±0.14 | 1.07±0.18 |  |  | 1.05±0.04 | 0.73±0.09* |  |  | 0.68±0.08 | 0.98±0.05* | |  |
| Glutamate | 1±0.25 | 1.86±0.39 |  |  | **5.85±0.45** | **6.87±1.46** |  |  | 1.38±0.30 | 1.27±0.29 | |  |
| Glutamine | 1±0.28 | 0.64±0.30 |  |  | 1.05±0.19 | 1.18±0.42 |  |  | **2.27±0.29** | **3.22±0.56** | |  |
| Glycine | 1±0.07 | 1.37±0.05* |  |  | **56.51±4.62** | **79.66±9.89*** |  |  | **8.05±0.88** | **8.34±2.09** | |  |
| Isoleucine | 1±0.05 | 1.07±0.04 |  |  | **64.58±2.20** | **58.38±7.21** |  |  | **16.24±1.87** | 12.69±6.28 | |  |
| Lysine | 1±0.09 | 1.21±0.11 |  |  | **81.72±4.81** | **67.33±10.05** |  |  | **23.07±2.32** | 18.14±7.94 | |  |
| Methionine | 1±0.12 | 1.02±0.04 |  |  | **9.61±0.75** | **10.24±2.19** |  |  | **3.94±0.57** | 3.61±1.28 | |  |
| Ornithine | 1±0.18 | 0.84±0.06 |  |  | **7.71±0.71** | **5.94±0.99** |  |  | 1.10±0.12 | 1.36±0.23 | |  |
| Phenylalanine | 1±0.12 | 1.05±0.07 |  |  | **61.98±3.71** | **51.78±7.20** |  |  | **14.07±1.85** | 11.96±6.22 | |  |
| Proline | 1±0.15 | 1.13±0.13 |  |  | **15.64±1.25** | **22.99±1.78*** |  |  | **13.70±3.89** | **11.95±2.88** | |  |
| Serine | 1±0.08 | 1.02±0.04 |  |  | **23.75±1.96** | **24.11±3.48** |  |  | **4.24±1.38** | **3.16±0.51** | |  |
| Threonine | 1±0.06 | 1.16±0.07 |  |  | **3.88±0.19** | **3.94±0.59** |  |  | **1.60±0.06** | 1.63±0.21 | |  |
| Tryptophan | 1±0.08 | 0.86±0.09 |  |  | **3.66±0.75** | 5.07±1.71 |  |  | **3.19±0.77** | 3.69±1.93 | |  |
| Tyrosine | 1±0.13 | 1.08±0.11 |  |  | **67.80±6.57** | **51.06±11.72** |  |  | **27.17±3.85** | 24.75±12.79 | |  |
| Valine | 1±0.03 | 1.04±0.03 |  |  | **24.39±0.71** | **21.64±2.16** |  |  | **7.80±0.69** | 5.83±2.46 | |  |
| **Organic acids** |  |  |  |  |  |  |  |  |  |  | |  |
| Citrate | 1±0.13 | 1.62±0.19* |  |  | **1.47±0.12** | 1.31±0.10 |  |  | 1.26±0.08 | **0.84±0.13*** | |  |
| Dehydroascorbate | 1±0.17 | 1.02±0.16 |  |  | **0.48±0.03** | 0.74±0.12* |  |  | 1.33±0.15 | 0.86±0.06* | |  |
| Fumarate | 1±0.05 | 1.30±0.09* |  |  | 0.98±0.06 | 1.20±0.19 |  |  | **1.85±0.11** | 1.35±0.12* | |  |
| Galactonate | 1±0.10 | 1.02±0.08 |  |  | **1.67±0.23** | 3.03±0.75 |  |  | **2.14±0.14** | **1.82±0.19** | |  |
| 2-oxo-Glutarate | 1†* | n.d. |  |  | 1.72±0.15 | 1.89±0.35 |  |  | 0.98±0.14 | 0.85±0.15 | |  |
| Glycerate | 1±0.04 | 1.04±0.06 |  |  | **0.39±0.03** | **0.41±0.09** |  |  | **0.83±0.03** | 1.19±0.10* | |  |
| Malate | 1±0.12 | 1.55±0.16* |  |  | **1.48±0.10** | 2.11±0.29* |  |  | **2.81±0.25** | **3.04±0.33** | |  |
| Nicotinate | 1±0.11 | 1.01±0.08 |  |  | 0.95±0.14 | 1.11±0.18 |  |  | 0.94±0.14 | 1.10±0.17 | |  |
| Pyruvate | 1±0.08 | 1.01±0.07 |  |  | **0.68±0.09** | **0.72±0.07** |  |  | **0.58±0.03** | **0.69±0.05** | |  |
| Succinate | 1±0.11 | 1.14±0.09 |  |  | **7.76±0.36** | **5.77±0.89** |  |  | **3.06±0.32** | 2.47±0.74 | |  |
| **Sugar and sugars alcohols** |  |  |  |  |  |  |  |  |  |  | |  |
| Erythritol | 1±0.11 | 2.59±0.27* |  |  | 3.04±1.08 | 2.78±0.40 |  |  | **2.51±0.26** | 3.16±0.66 | |  |
| Fructose | 1±0.07 | 0.95±0.12 |  |  | **5.88±0.47** | **2.83±0.96*** |  |  | **31.96±2.48** | **15.72±3.04*** | |  |
| Galactinol | 1±0.17 | 0.84±0.23 |  |  | 0.96±0.10 | 1.79±0.37 |  |  | **8.44±0.10** | **7.58±0.37** | |  |
| Glucose | 1±0.17 | 1.09±0.17 |  |  | **3.40±0.21** | **3.14±0.62** |  |  | **168.6±8.62** | **101.92±20.9*** | |  |
| Glycerol | 1±0.10 | 1.19±0.13 |  |  | **2.05±0.15** | 3.26±0.84 |  |  | 0.98±0.11 | 1.31±0.28 | |  |
| Glycerol-3-P | 1† | n.d. |  |  | 6.10±0.82 | 9.71±2.99 |  |  | n.d. | n.d. | |  |
| myo-Inositol | 1±0.05 | 1.03±0.14 |  |  | **1.28±0.08** | 2.19±0.40 |  |  | **2.25±0.08** | **2.11±0.11** | |  |
| Maltose | 1±0.13 | 1.69±0.32* |  |  | **5.10±0.76** | **5.49±1.11** |  |  | **7.83±2.05** | 7.91±3.10 | |  |
| Raffinose | 1±0.15 | 0.90±0.19 |  |  | **1.98±0.17** | **2.24±0.41** |  |  | **2.03±0.38** | **2.00±0.34** | |  |
| Sucrose | 1±0.05 | 1.14±0.08 |  |  | **10.99±0.33** | **11.44±1.90** |  |  | **2.77±0.63** | 2.40±0.62 | |  |
| Trehalose | 1±0.11 | 1.09±0.14 |  |  | 1.21±0.09 | 2.01±0.49 |  |  | **1.68±0.18** | 2.18±0.69 | |  |
| Xylose | 1±0.08 | 1.03±0.08 |  |  | 1.13±0.07 | 1.17±0.14 |  |  | **5.72±0.63** | 4.44±1.59 | |  |
| **Others metabolites** |  |  |  |  |  |  |  |  |  |  | |  |
| beta-Alanine | 1±0.10 | 1.09±0.05 |  |  | **8.27±0.50** | **9.24±1.15** |  |  | **2.98±0.28** | 3.07±0.86 | |  |
| Phosphoric acid | 1±0.49 | 0.60±0.09 |  |  | 1.08±0.23 | 0.98±0.21 |  |  | 4.14±3.50 | 0.58±0.08 | |  |
| 4-hydroxy-Proline | 1±0.08 | 1.09±0.06 |  |  | **4.15±0.25** | **5.30±0.94** |  |  | **3.36±0.27** | **4.01±0.56** | |  |
| Putrescine | 1±0.15 | 2.31±0.48* |  |  | **4.94±0.62** | **6.70±0.97** |  |  | **8.94±1.27** | **7.64±1.85** | |  |
| Spermidine | 1±0.12 | 1.19±0.44 |  |  | **2.72±0.51** | **5.49±1.05*** |  |  | **5.43±0.84** | 6.52±1.63 | |  |
| Urea | 1±0.30 | 1.04±0.12 |  |  | **5.14±1.09** | **4.84±0.99** |  |  | 1.01±0.14 | 0.87±0.44 | |  |
