# Supplementary material for: The Lack of Alternative Oxidase 1a Restricts in vivo Respiratory Activity and Stress-Related Metabolism for Leaf Osmoprotection and Redox Balancing Under Sudden Acute Water and Salt Stress in Arabidopsis thaliana
Source: Front Plant Sci. 2022 May 17;13:833113. doi: 10.3389/fpls.2022.833113 (PMC9152546; doi:10.3389/fpls.2022.833113)
Supplement: Supplementary file 3 [file Table_3.DOCX]

**Supplemental Table 3.** Absolute metabolite levels in leaves of WT and *AOX1a* plants under control conditions and after 1 day of severe (300 mM) NaCl and Mannitol treatments as measured by GC-MS (see material and methods for details). Data is presented as means ± SE for 4 to 6 biological replicates. Bold numbers denote significant differences (*P* < 0.05) to the control condition in each genotype separately. Asterisks denote significant differences (*P* < 0.05) between WT and *aox1a* plants in each experimental condition. †Denotes metabolites detected only in one replicate in WT at control conditions. ‘n.d.’ denotes cases for not detected metabolites.

|  | **Control** | |  | | **NaCl** | |  | | | **Mannitol** | |  |
| --- | --- | --- | --- | --- | --- | --- | --- | --- | --- | --- | --- | --- |
| Metabolite | *WT* | *AOX1a* |  |  | *WT* | *AOX1a* | |  |  | *WT* | *AOX1a* |  |
| **Amino acids** |  |  |  |  |  |  | |  |  |  |  |  |
| Alanine | 0.509±0.146 | 0.479±0.120 |  |  | **1.51±0.305** | 1.64±0.410 | |  |  | 0.919±0.222 | **1.10±0.150** |  |
| Asparagine | 0.145±0.022 | 0.130±0.009 |  |  | **0.469±0.037** | **0.604±0.037*** | |  |  | **0.299±0.051** | 0.285±0.068 |  |
| Aspartate | 0.082±0.011 | 0.088±0.015 |  |  | 0.087±0.004 | 0.060±0.007* | |  |  | 0.056±0.006 | 0.081±0.004* |  |
| Glutamate | 0.316±0.079 | 0.588±0.122 |  |  | **1.85±0.141** | **2.17±0.461** | |  |  | 0.437±0.095 | 0.402±0.093 |  |
| Glutamine | 0.364±0.102 | 0.233±0.108 |  |  | 0.381±0.070 | 0.429±0.153 | |  |  | **0.826±0.105** | **1.17±0.203** |  |
| Glycine | 0.183±0.012 | 0.252±0.009* |  |  | **7.14±0.580** | **10.01±1.24*** | |  |  | **1.01±0.111** | **1.05±0.263** |  |
| Isoleucine | 0.022±0.001 | 0.023±0.001 |  |  | **1.41±0.048** | **1.28±0.158** | |  |  | **0.355±0.041** | 0.278±0.137 |  |
| Lysine | 0.013±0.001 | 0.015±0.001 |  |  | **1.03±0.061** | **0.852±0.127** | |  |  | **0.292±0.029** | 0.229±0.100 |  |
| Methionine | 0.010±0.001 | 0.010±0.000 |  |  | **0.092±0.007** | **0.099±0.021** | |  |  | **0.038±0.005** | 0.035±0.012 |  |
| Ornithine | 0.008±0.001 | 0.006±0.000 |  |  | **0.059±0.005** | **0.045±0.008** | |  |  | 0.008±0.001 | 0.010±0.002 |  |
| Phenylalanine | 0.018±0.002 | 0.019±0.001 |  |  | **1.12±0.067** | **0.933±0.130** | |  |  | **0.254±0.033** | 0.216±0.112 |  |
| Proline | 0.150±0.022 | 0.170±0.020 |  |  | **2.34±0.188** | **3.44±0.266*** | |  |  | **2.05±0.582** | **1.79±0.431** |  |
| Serine | 0.875±0.067 | 0.892±0.036 |  |  | **4.22±0.348** | **4.28±0.618** | |  |  | **0.752±0.245** | **0.561±0.091** |  |
| Tryptophan | 0.008±0.001 | 0.007±0.001 |  |  | **0.029±0.006** | 0.040±0.013 | |  |  | **0.025±0.006** | 0.029±0.015 |  |
| Tyrosine | 0.004±0.001 | 0.004±0.000 |  |  | **0.271±0.026** | **0.204±0.047** | |  |  | **0.109±0.015** | 0.099±0.051 |  |
| Valine | 0.082±0.002 | 0.086±0.002 |  |  | **2.01±0.0.059** | **1.78±0.178** | |  |  | **0.643±0.057** | 0.480±0.203 |  |
| **Organic acids** |  |  |  |  |  |  | |  |  |  |  |  |
| Citrate | 0.022±0.003 | 0.035±0.004* |  |  | **0.032±0.003** | 0.028±0.002 | |  |  | 0.027±0.002 | **0.018±0.003*** |  |
| Fumarate | 4.68±0.269 | 4.95±0.180* |  |  | 5.52±0.364 | 6.77±1.06 | |  |  | **10.41±0.619** | 7.59±0.649* |  |
| 2-oxo-Glutarate | 0.024†* | n.d. |  |  | 0.040±0.004 | 0.044±0.008 | |  |  | 0.023±0.003 | 0.020±0.004 |  |
| Glycerate | 1.49±0.062 | 1.55±0.083 |  |  | **0.584±0.050** | **0.607±0.131** | |  |  | **1.23±0.040** | 1.77±0.145* |  |
| Malate | 0.365±0.044 | 0.567±0.058* |  |  | **0.540±0.038** | 0.772±0.105* | |  |  | **1.03±0.092** | **1.11±0.121** |  |
| Pyruvate | 1.23±0.096 | 1.24±0.090 |  |  | **0.840±0.112** | **0.890±0.092** | |  |  | **0.710±0.041** | **0.845±0.061** |  |
| Succinate | 0.033±0.004 | 0.037±0.003 |  |  | **0.254±0.012** | **0.189±0.029** | |  |  | **0.100±0.011** | 0.081±0.024 |  |
| **Sugar and sugars alcohols** |  |  |  |  |  |  | |  |  |  |  |  |
| Erythritol | 0.014±0.001 | 0.035±0.004* |  |  | 0.041±0.015 | 0.038±0.005 | |  |  | **0.034±0.003** | 0.043±0.009 |  |
| Fructose | 0.129±0.009 | 0.123±0.015 |  |  | **0.759±0.060** | **0.365±0.123*** | |  |  | **4.12±0.320** | **2.03±0.393*** |  |
| Galactinol | 0.039±0.007 | 0.033±0.009 |  |  | 0.038±0.004 | 0.070±0.015 | |  |  | **0.330±0.039** | **0.296±0.041** |  |
| Glucose | 0.335±0.059 | 0.364±0.056 |  |  | **1.14±0.071** | **1.05±0.207** | |  |  | **56.49±2.89** | **34.15±7.00*** |  |
| Glycerol | 0.101±0.010 | 0.120±0.013 |  |  | **0.206±0.015** | 0.329±0.085 | |  |  | 0.099±0.011 | 0.132±0.028 |  |
| Glyerol-3-P | 0.014† | n.d. |  |  | 0.088±0.012 | 0.140±0.043 | |  |  | n.d. | n.d. |  |
| *myo*-Inositol | 0.634±0.035 | 0.653±0.087 |  |  | **0.811±0.049** | 1.39±0.256 | |  |  | **1.43±0.053** | **1.34±0.067** |  |
| Raffinose | 0.337±0.051 | 0.303±0.064 |  |  | **0.666±0.058** | **0.754±0.139** | |  |  | **0.682±0.129** | **0.674±0.113** |  |
| Sucrose | 1.74±0.084 | 1.98±0.140 |  |  | **19.11±0.580** | **19.90±3.30** | |  |  | **4.82±1.103** | 4.17±1.08 |  |
| Trehalose | 0.011±0.001 | 0.012±0.001 |  |  | 0.013±0.001 | 0.022±0.005 | |  |  | **0.018±0.002** | 0.024±0.007 |  |
| Xylose | 0.040±0.003 | 0.041±0.003 |  |  | 0.046±0.003 | 0.047±0.006 | |  |  | **0.230±0.025** | 0.178±0.064 |  |
| **Others metabolites** |  |  |  |  |  |  | |  |  |  |  |  |
| beta-Alanine | 0.015±0.002 | 0.016±0.001 |  |  | **0.122±0.007** | **0.136±0.017** | |  |  | **0.044±0.004** | 0.045±0.013 |  |
| Phosphoric acid | 0.011±0.005 | 0.007±0.001 |  |  | 0.012±0.003 | 0.011±0.002 | |  |  | 0.046±0.039 | 0.006±0.001 |  |
| Putrescine | 0.029±0.004 | 0.068±0.014* |  |  | **0.145±0.018** | **0.197±0.029** | |  |  | **0.263±0.038** | **0.225±0.055** |  |
