# Supplementary material for: The Lack of Alternative Oxidase 1a Restricts in vivo Respiratory Activity and Stress-Related Metabolism for Leaf Osmoprotection and Redox Balancing Under Sudden Acute Water and Salt Stress in Arabidopsis thaliana
Source: Front Plant Sci. 2022 May 17;13:833113. doi: 10.3389/fpls.2022.833113 (PMC9152546; doi:10.3389/fpls.2022.833113)
Supplement: Supplementary file 4 [file Table_4.DOCX]

**Supplemental Table 4.** Primers used in the qPCR analyses performed in this study.

| **Target** | **Gene name** | **Locus** | **Forward primer** | **Reverse Primer** |
| --- | --- | --- | --- | --- |
| AtUbqC | Ubiquitine C | AT5G25760 | TCAAATGGACCGCTCTTATC | CACAGACTGAAGCGTCCAAG |
| AtUCP1 | Uncoupling Mitochondrial Protein 1 | AT3G54110 | TCTGCTCTTGCTGGTGATGT | TACCCAGTGCACCTGTTGTC |
| At UCP2 | Uncoupling Mitochondrial Protein 2 | AT5G58970 | GGATTTCAAACCAAGGATCG | AGCGCACTAACTCCTTCCAG |
| AtNDA1 | Alternative NAD(P)H Dehydrogenase A1 | AT1G07180 | CTCCGTGAGAGCAAGGAAGG | GGCGAAGTGGAGGGGATATG |
| AtNDA2 | Alternative NAD(P)H Dehydrogenase A2 | AT2G29990 | CGAGAGCAAGGACGCAAAAG | CAGTAGGCCGAGATTGAGAC |
| AtNDB2 | Alternative NAD(P)H Dehydrogenase B2 | AT4G05020 | ACTGACTCTCAAAGAGTTCC | CCGATTTGAACTCTTCGATC |
| AtNDB3 | Alternative NAD(P)H Dehydrogenase B3 | AT4G21490 | GGTGAGTAGCCAAAGACGTG | GAAGATCGGTAATGCCATGC |
| AOX1a | Alternative oxidase 1a | AT3G22370 | CCGATTTGTTCTTCCAGAGG | GCGCTCTCTCGTACCATTTC |
| AOX1b | Alternative oxidase 1b | AT3G22360 | CTTTTCTTCCAGAGGCGGTA | TTAGGTTTCGCGACTTCCAT |
| AOX1c | Alternative oxidase 1c | AT3G27620 | CCGATCTTTTCTTCCAGAGG | TGGGAGAGATTATGTATCCGATT |
| AOX1d | Alternative oxidase 1d | AT1G32350 | GCCATGGGATTCATACAAAC | CACATGTGCTTCCTCTGAAAA |
| AOX2 | Alternative oxidase 2 | AT5G64210 | TGGGAAGATCGAGAATGTTG | TCCTTGATTGCGAATGTCAG |
