# Supplementary figures and images for: The Lack of Alternative Oxidase 1a Restricts in vivo Respiratory Activity and Stress-Related Metabolism for Leaf Osmoprotection and Redox Balancing Under Sudden Acute Water and Salt Stress in Arabidopsis thaliana
Source: Front Plant Sci. 2022 May 17;13:833113. doi: 10.3389/fpls.2022.833113 (PMC9152546; doi:10.3389/fpls.2022.833113)

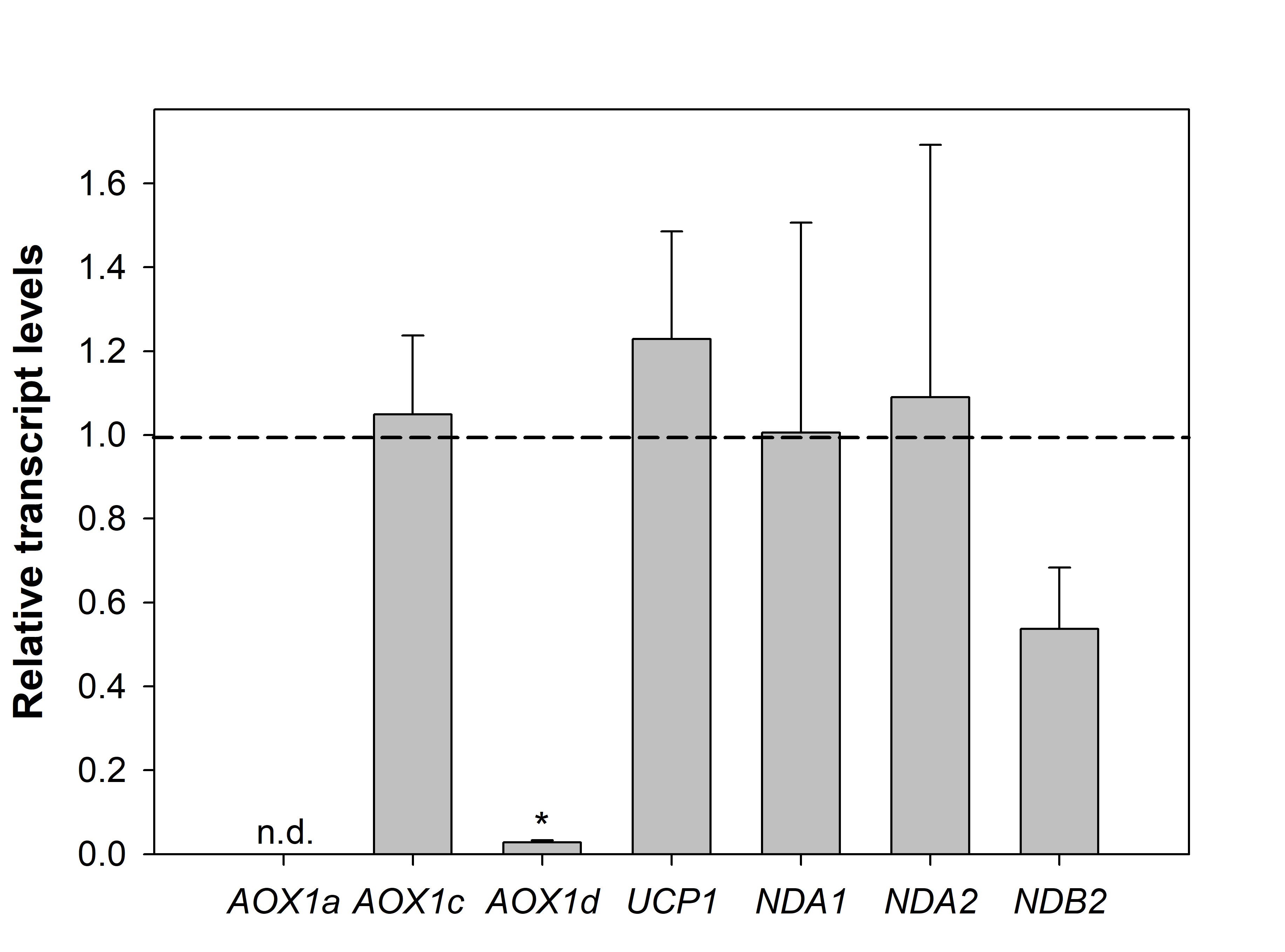

Supplement: Supplementary Figure 1 — Gene expression analyses of alternative respiratory components under control conditions. Transcript levels of genes encoding alternative oxidases, uncoupling proteins and alternative NAD(P)H dehydrogenases were determined by qPCR analyses (see Materials and Methods for details) in leaves of wild-type Col 0 (WT) and aox1a A. thaliana plants as described in Figure 5A, 6. Data is shown as fold-changes relative to WT (i.e., all WT values were set to 1, which is denoted by the dashed line). Primers used and gene information can be found in Supplementary Table 4. Values are means ± SE of 5–6 replicates and asterisks denote significant differences (P < 0.05) to the WT for each gene expression analysis. n.d. (not detected). [file Image_1.JPEG]
